# Abalone genomics reveals an ancient asymmetry axis and the multifunctionality of the mantle

**DOI:** 10.1126/sciadv.aeb8392

**Published:** 2026-09-04

**Authors:** Xiaotong Wang, Chunhui Li, Peidong Xin, Meiwei Zhang, Baoyu Huang, Tiantian Chen, Siyi Li, Yijing Han, Lei Wei, Xuekai Zhang, Yaqiong Liu, Chenglong Zhu, Wenjie Xu, Zhiyong Yue, Wenshi Xue, Wenchao Yu, Peng Zhang, Lingling Li, Mengqiang Yuan, Deyang Tian, Jiyan Qi, Qiang Qiu, Kun Wang, Ximing Guo, Chenguang Feng

**Affiliations:** ^1^ School of Fisheries, Ludong University, Yantai, 264025, China.; ^2^ Shaanxi Key Laboratory of Qinling Ecological Intelligent Monitoring and Protection, School of Life Science and Technology, Northwestern Polytechnical University, Xi’an, 710072, China.; ^3^ Yantai Institute, China Agricultural University, Yantai, 264670, China.; ^4^ Key Laboratory of Natural Anti-aging Product Mining and Biosynthesis of Shaanxi Higher Education Institutes, Applied Research Institute of Life Sciences, Xi’an International University, Xi’an, 710077, China.; ^5^ Laizhou LiYang Aquatic Development Co., Ltd., Yantai, 261441, China.; ^6^ Haskin Shellfish Research Laboratory, Department of Marine and Coastal Sciences, Rutgers University, Port Norris, NJ 08349, USA.

## Abstract

Gastropod diversification represents one of the most spectacular evolutionary radiations, underpinned by a fundamentally asymmetric body plan and diverse pigmentation patterns. To understand the genetic underpinnings of these cardinal traits, we constructed a chromosome-level genome assembly for the rainbow abalone, *Haliotis iris*. Integrating comparative genomics, histoembryology, and molecular assays, we identify a conserved regulatory association between the long non-coding RNA *lncRNA1* and *pitx* that is associated with asymmetric mantle development. In *H. iris*, this association is supported by chromatin-contact evidence and exploratory data suggesting a possible miRNA-associated post-transcriptional component. Furthermore, we identify a *wnt-mitf-tyr* framework for melanogenesis and show that the mantle is the primary site of melanin synthesis. Concurrently, we identify mantle-enriched *prestin* genes that are vibration-responsive, consistent with a possible sensory specialization of the mantle. These findings provide molecular insight into asymmetry, pigmentation, and mantle multifunctionality in gastropods.

## INTRODUCTION

Gastropods, with their unparalleled radiation into over 80,000 extant species, represent a pinnacle case of morphological and ecological diversification within the Mollusca (*1*, *2*). Their hallmark is an asymmetric body plan forged by torsion and subsequent shell coiling, a developmental process that reorients the visceral mass relative to the head and foot, fundamentally shaping their morphology and ecological adaptations (*3*, *4*). This asymmetry, along with melanin-based pigmentation critical for photoprotection, camouflage, and signaling, underpins their phenotypic diversity (*5*–*7*). These features, asymmetry and pigmentation, are not merely curiosities but windows into the genetic and molecular mechanisms that have driven the diversification of the gastropod clade.

Despite substantial advances in molluscan genomics (*8*), key gaps remain in our understanding of these core gastropod traits. The molecular basis of organ asymmetry remains elusive; while *pitx*, known to determine left-right asymmetry in bilaterians (*9*, *10*), has been reported to be associated with coiling in gastropods (*4*, *11*, *12*), its regulatory architecture in gastropods remains inadequately comprehended. Similarly, melanin synthesis, well-described in vertebrates and a few invertebrates through the gene *tyr* (*13*–*16*), is understudied in gastropods, leaving its pathway conservation and regulation unclear (*17*, *18*).

**Fig. 1.**
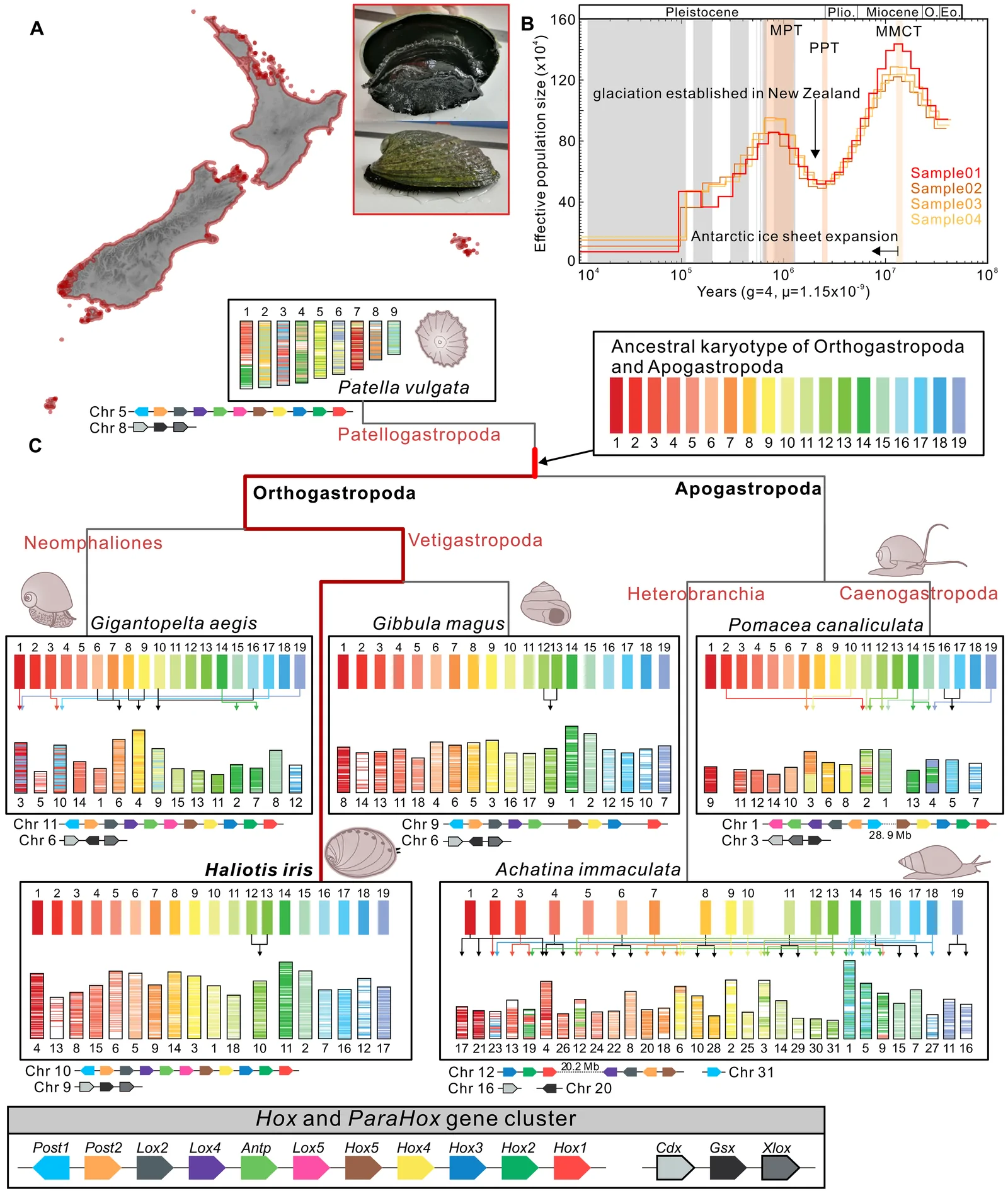
Ecological distribution and evolutionary analyses of rainbow abalone *H. iris*. (**A**) Morphology and geographical distribution. Note that the rainbow abalone image in (A) is also presented in Fig. 6C for mantle localization. (**B**) PSMC-based reconstruction of historical effective population size in four *H. iris* individuals. The *x* axis indicates time before present in years, scaled using a generation time (g) and a per-site per-generation substitution/mutation rate (μ). Orange bars indicate major paleoclimatic events with annotations; gray bars represent Pleistocene glacial periods. (**C**) Reconstruction and comparison of ancestral proto-chromosomal fragments and *Hox/ParaHox* gene organization in six representative gastropod genomes. The colored bars represent inferred ancestral proto-chromosomal fragments of Orthogastropoda and Apogastropoda, and their distribution across extant chromosomes. The lower schematic summarizes the chromosomal organization of *Hox* and *ParaHox* genes.

The rainbow abalone, *Haliotis iris*, offers a unique system for studying gastropod asymmetry and melanogenesis. A hallmark of the Haliotidae is a partial, ∼90-degree detorsion that follows the canonical 180-degree gastropod torsion (*19*, *20*). This process directly results in an anterior mantle cleft with asymmetric lobes (right > left), a defining morphological synapomorphy that differs from most gastropods with a unified mantle (*20*, *21*). Complementing this morphological novelty is its suitability for investigating melanogenesis. The species exhibits a striking eumelanin-based black phenotype (Fig. 1A) (*22*), yet includes the natural variation of unpigmented regions in some individuals (see below), providing a tractable system for genetic dissection. As an ecologically significant species in New Zealand’s intertidal zones (Fig. 1A) and one of the top three abalone species in global trade (*23*–*25*), *H. iris* combines profound evolutionary interest with substantial economic and cultural relevance.

Leveraging a high-quality, chromosome-level genome assembly of *H. iris* and comprehensive comparative analyses across gastropods, we delineated genomic features consistent with its evolutionary conservatism. We then place *H. iris* in a comparative genomic and evolutionary context, dissect the molecular basis of developmental asymmetry and pigmentation, and finally use these insights to refine the molecular understanding of mantle multifunctionality. In addition, we uncover a severe demographic bottleneck in *H. iris* over the last 110,000 years, resulting in a small effective population size and underscoring the need for conservation of this ecologically and economically important species.

## RESULTS AND DISCUSSION

### Genome information

A chromosome-level *H. iris* genome assembly was obtained using a combination of Oxford Nanopore (∼148.6× coverage), Illumina (∼178.3× coverage), and Hi-C (∼186.4× coverage) sequencing reads (table S1). The final assembly spanned 1,150.57 Mb, with a scaffold N50 of 66.58 Mb and a contig N50 of 12.77 Mb (Table 1). A total of 99.1% of the assembled bases were anchored to 18 pseudo-chromosomes (fig. S1), consistent with the expected haploid chromosome number and genome size (*26*, *27*) (fig. S2). The BUSCO assessment indicated high completeness and accuracy, with 96.8% of metazoan conserved core genes covered (table S2) and a single-base error rate of less than 0.0627 per megabase (Table 1). Genome annotation identified 20,994 protein-coding genes and 566.94 Mb of repetitive sequences, with the latter accounting for 49.3% of the genome (Table 1; table S3). The protein-coding genes included 98.7% of metazoan BUSCOs (table S2), and 91.18% of these genes were supported by transcript evidence (17,626 genes) or homology in public databases (18,095 genes) (table S4). The quality of the *H. iris* genome surpasses that of most published molluscan genomes and represents the highest-quality genome for the Haliotidae family to date (fig. S3), providing a robust foundation for subsequent analyses.

**Table 1.** Assembly statistics of the rainbow abalone genome.

Feature	Statistics
Assembly size (Mb)	1,150.57
Scaffold N50 (Mb)	66.58
Contig N50 (Mb)	12.77
Longest scaffold (Mb)	84.81
Longest contig (Mb)	40.58
Haploid number of *pseudo*-chromosomes	18
Chromosome bases/total length (%)	99.09
Base error rate in assembly (per Mb)	0.0627
Repeats (Mb)	566.94
Protein-coding genes	20,994
BUSCO of assembly (%)	96.8
BUSCO of Protein-coding genes (%)	98.7

### Demographic history and chromosome evolution

Phylogenetic analysis revealed that *H. iris* diverged from its sibling species, *H. rufescens*, around 39.9 Ma (95% CI: 23.19–56.36 Ma; fig. S4). We reconstructed the demographic history of rainbow abalone over the past 40 Ma by estimating effective population size (Fig. 1B). Over this period, the population experienced three major fluctuations and one severe bottleneck. It declined from its peak during the Middle Miocene Climate Transition (MMCT) (*28*, *29*) around 14.55–12.16 Ma; rebounded after the warmer Plio-Pleistocene Transition (PPT) (*30*) from 2.65–1.96 Ma; dropped again after the cooler Mid-Pleistocene Transition (MPT) (*31*) around 0.93–0.71 Ma; and underwent a severe contraction from ∼0.11 Ma, coinciding with the onset of the Würm glaciation (*32*), which resulted in a severe bottleneck with a small effective population size of 7.47–16.89 (×10^4^). Re-sequencing of three additional rainbow abalone individuals validated these demographic dynamics (Fig. 1B).

Chromosome evolution was investigated by comparing the karyotypes of six representative gastropod species: *Patella vulgata*, *Gigantopelta aegis*, *H. iris*, *Gibbula magus*, *Achatina immaculata*, and *Pomacea canaliculata* (Fig. 1C). Reconstruction of the ancestral karyotype for Orthogastropoda and Apogastropoda (2n = 38; table S5) revealed that while chromosomal fusions and fissions are frequent across gastropods, the Vetigastropoda lineage, which includes *H. iris*, exhibits remarkable karyotypic conservation with only one inferred proto-chromosome fusion event. This broader pattern was further supported by an expanded comparison across 18 chromosome-level gastropod genomes, including three additional abalones (*Haliotis asinina*, *Haliotis ovina*, and *H. tuberculata*) and several other Vetigastropods, which showed that most Vetigastropod species retain relatively conservative ancestral chromosomal architectures despite lineage-specific deviations (fig. S5). Furthermore, we examined the arrangement of *Hox* and *ParaHox* gene clusters, finding that *H. iris* has retained the conserved ancestral configuration for both (Fig. 1C and fig. S5). Taken together, the abalone preserves some ancient genetic features, representing an archetypal, evolutionarily conserved lineage of gastropods.

An intriguing correlation emerges in which lineages with few karyotypic changes (e.g., *H. iris* and *G. magus*) are typically associated with higher dispersal potential, whereas lineages with extensive karyotypic rearrangements are often terrestrial, freshwater, hydrothermal-vent, or otherwise more dispersal-limited taxa (table S6). One possible explanation is that reduced dispersal increases population subdivision and local retention, thereby facilitating the fixation of chromosomal mutations. This interpretation is consistent with observations in scallops (*33*, *34*), where the broadcast-spawning *Patinopecten yessoensis*, a species with wide larval dispersal, retains a highly conserved karyotype similar to the ancestral bilaterian condition. The expanded Vetigastropod comparison further refines this pattern (table S6): although *Tricolia pullus* and *H. tuberculata* show comparatively faster karyotypic change (fig. S5), these cases do not necessarily violate the trend. Rather, *T. pullus* combines patchy algal-associated habitats with a very short pelagic phase (*35*), and *H. tuberculata* shows marked spatial population structuring and local retention despite its broadcast-spawning life history (*36*), both of which are consistent with more restricted realized connectivity. Further studies are needed to fully understand the mechanisms and evolutionary consequences of karyotypic evolution.

### A *lncRNA1-pitx* regulatory association is linked to mantle asymmetry

**Fig. 2.**
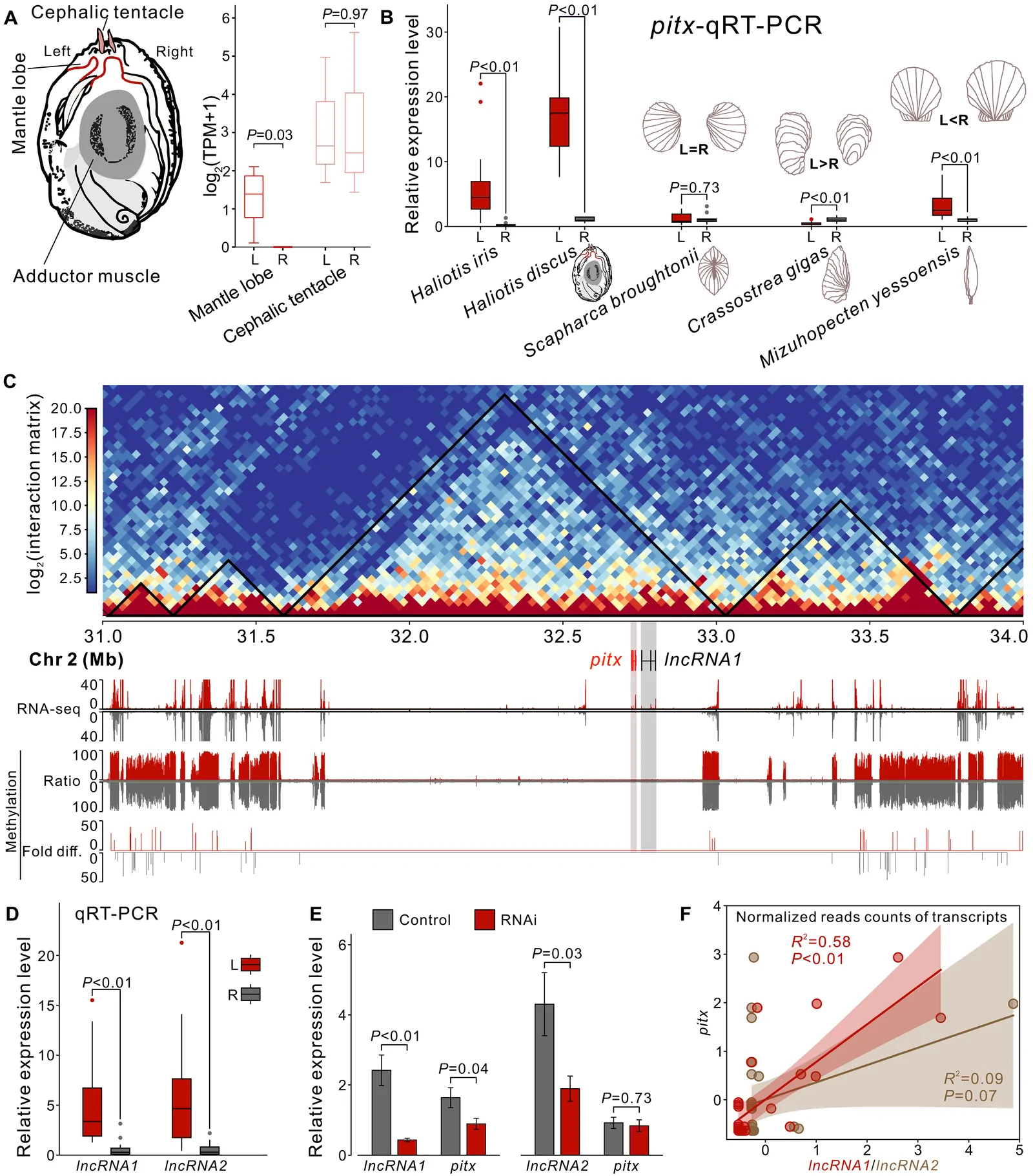
Asymmetric expression of *pitx* and lncRNAs in paired mantle lobes. (**A**) Dorsal schematic of the shell-less abalone showing the asymmetric mantle lobes and symmetric cephalic tentacles, together with RNA-seq-based *pitx* expression in paired left and right samples. *n* = 4 for mantle lobes and *n* = 3 for cephalic tentacles. (**B**) qRT-PCR validation of *pitx* expression in paired left and right mantle lobes from *H. iris* and four additional mollusks. Schematics above the plots indicate the relative left–right mantle morphology for each species. Relative expression was calculated by the 2^-ΔΔCt^ method. *n* = 16 for *H. iris*, *n* = 8 for *Haliotis discus*, *n* = 9 for *S. broughtonii*, *n* = 14 for *C. gigas*, and *n* = 8 for *M. yessoensis*. (**C**) Chromatin interactions, transcripts, and DNA methylation profiles across the Chr2:31–34 Mb region surrounding *pitx*; black lines indicate TADs; *pitx* and *lncRNA1* models are shown in red and black, respectively; RNA-seq and methylation tracks show left side in red and right side in gray. (**D**) qRT-PCR comparing the expression of two lncRNAs in left *vs* right mantle lobes of rainbow abalone. Relative expression was calculated by the 2^-ΔΔCt^ method, *n* = 16 per group. (**E**) qRT-PCR results showing that only *lncRNA1* knockdown reduces *pitx* expression. Data are mean ± SEM. Significance was assessed using an independent-samples *t* test, with *n* = 9 per group. (**F**) Linear regression between *pitx* expression and *lncRNA1* or *lncRNA2* expression across *H. iris* tissues, based on normalized transcript read counts from the RNA-seq samples listed in table S1. Shaded regions indicate the confidence intervals. *P* values are indicated in the corresponding panels. Significance was assessed using a paired *t* test for [(A), (B), and (D)].

The abalone mantle is a classic example of organ asymmetry in a bilaterally symmetric animal, with the right side being larger than the left (Fig. 2A). To identify candidate regulators associated with this asymmetry, we compared transcriptomes of paired left and right mantle lobes and, as a symmetric control, paired left and right cephalic tentacles. Because the left and right mantle lobes are corresponding regions of the same organ, rather than distinct tissues, their global transcriptomic profiles were highly similar and did not show strong separation by PCA (fig. S6). We therefore focused on robust left–right-biased candidates. No genes were differentially expressed between the symmetric cephalic tentacles (Fig. 2A and table S7). In contrast, *pitx* emerged as the most prominent left-biased protein-coding candidate associated with the asymmetric mantle lobes, and this signal was retained after reanalysis excluding the relatively separated paired mantle sample shown in the PCA plot (Fig. 2A; fig. S6; table S8). Its expression was high in the smaller left lobe but low or nearly undetectable in the larger right lobe (Fig. 2, A and B; fig. S7; tables S8 and S9). This left-biased expression was also observed in the sibling species *Haliotis discus* (Fig. 2B and table S10). Across several bivalve species, *pitx* similarly showed higher expression on the smaller side of the mantle, except for *Scapharca broughtonii*, where the mantle lobes are of comparable size (Fig. 2B and table S10). These findings identify *pitx* as a strong candidate associated with asymmetric mantle growth.

Given that *pitx* was the most robust protein-coding candidate associated with mantle asymmetry, we next examined how its asymmetric expression might be regulated. In oysters, we previously found that asymmetric *pitx* expression correlated with differential DNA methylation in its upstream regulatory region (*37*). In *H. iris*, however, we observed extremely low methylation levels across a 150 kb region flanking *pitx* with no difference between the left and right sides (Fig. 2C and fig. S7), suggesting a distinct regulatory mechanism may be involved. A subsequent screen for non-coding RNAs (ncRNAs) identified two previously unannotated long ncRNAs (lncRNAs) with left-biased expression in the original mantle comparison, but not in the symmetric cephalic tentacles (table S11). Among them, *lncRNA1* is located 18.6 kb downstream of *pitx* and mirrors its left-biased expression pattern (Fig. 2, C and D; tables S9 and S11). *lncRNA2* is located on a different chromosome (Chr 12) and was also left-biased in the original dataset (Fig. 2D; tables S9 and S11), but the outlier-removal reanalysis (fig. S6 and table S11) and subsequent perturbation assays supported *lncRNA1* as the more robust candidate. Specifically, only *lncRNA1* knockdown consistently reduced *pitx* expression levels (Fig. 2E; fig. S8; table S12), supporting a directional regulatory relationship from *lncRNA1* to *pitx*. This conclusion was further supported by the positive correlation between *lncRNA1* and *pitx* expression across multiple tissues (Fig. 2F).

### Local regulatory context of the *lncRNA1-pitx* association in the asymmetric mantle

**Fig. 3.**
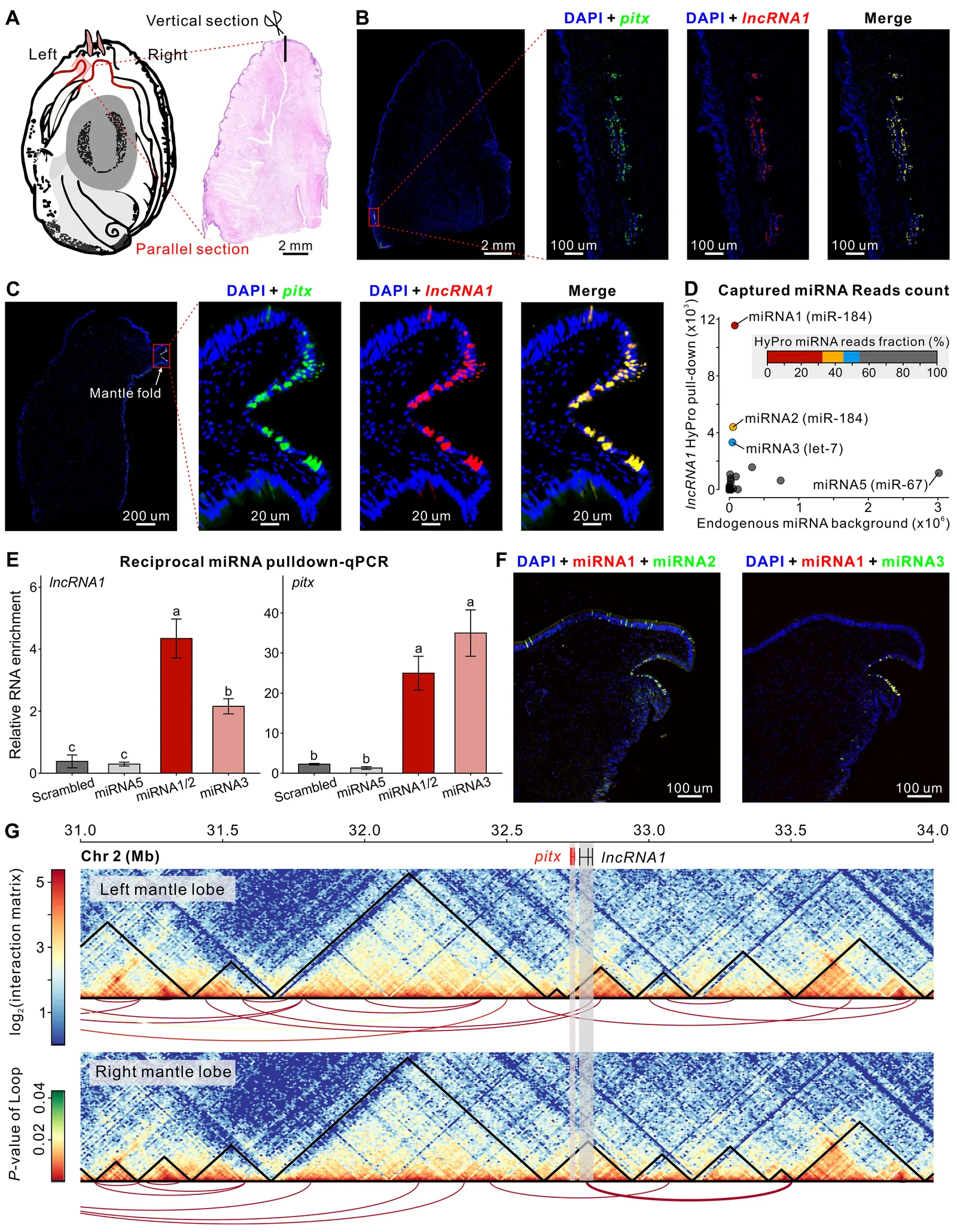
Spatial localization and molecular associations between *pitx* and *lncRNA1*. (**A**) Position diagram and parallel HE section of the left mantle lobe. (**B** and **C**) In situ hybridization results for the *pitx* and *lncRNA1* genes in the parallel (B) and vertical (C) sections of the left mantle lobe. DAPI marks nuclei. (**D**) HyPro-seq profiling of miRNAs captured by the *lncRNA1*-specific pulldown. Each dot represents a mature miRNA detected in the left mantle lobe. The x-axis indicates the endogenous miRNA background abundance, and the y-axis indicates the number of miRNA reads recovered in the *lncRNA1* HyPro pulldown. miRNA1 and miRNA2, two miR-184 isoforms with identical seed sequences, and miRNA3, a let-7 family miRNA (table S14), showed prominent recovery in the *lncRNA1* pulldown. (**E**) Reciprocal miRNA pulldown-qPCR analysis of *lncRNA1* and *pitx*. Biotinylated probes targeting miRNA1/2, mixed at a 1:1 ratio, or miRNA3 were used to capture endogenous miRNA-associated RNA complexes from crosslinked left mantle lobe lysates. Scrambled probes, consisting of a 1:1 mixture of two random probes, and a miRNA5 probe, targeting the most abundant background miRNA, were used as negative controls. Data are mean ± SEM; *n* = 3 per group. Significance was assessed using one-way ANOVA followed by Tukey’s HSD post hoc test. Different letters indicate significant differences at *P* < 0.05. (**F**) In situ hybridization results for the three miRNAs in the vertical sections of the left mantle lobe. DAPI marks nuclei. (**G**) Chromatin interactions in the Chr2:31–34 Mb region between left and right mantle lobes; black lines in the interaction plot indicate TADs; *pitx* and *lncRNA1* gene models shown in red and black, respectively; lines below the heatmap represent the DNA loops between Hi-C units, and line color indicates the corresponding *P* value.

To further investigate the regulatory relationship between *lncRNA1* and *pitx*, we performed a histological survey of the left anterior mantle lobe in *H. iris* (Fig. 3A). In situ hybridization showed that *pitx* and *lncRNA1* are present in the same mantle-fold cells and display overlapping cytoplasmic expression at the tissue level (Fig. 3, B and C). This highly restricted expression pattern likely explains the relatively low average abundance of *pitx* and the absence of broad downstream transcriptional signatures in the mantle comparison (table S8). Thus, *pitx* is best interpreted as a strong asymmetry-associated transcription-factor candidate, whose localized downstream effectors remain to be resolved by spatially resolved or single-cell approaches. The overlapping cytoplasmic expression of *lncRNA1* and *pitx* suggests that *lncRNA1* may regulate *pitx* at the post-transcriptional level, for example by helping maintain *pitx* mRNA abundance (*38*, *39*). To test whether small RNAs might be associated with this cytoplasmic regulatory module, we used HyPro-seq (*40*) with three *lncRNA1*-specific probes to capture *lncRNA1*-associated miRNAs (table S13). This analysis prioritized three candidate miRNAs: two closely related miR-184-3p candidates, miRNA1 and miRNA2, and one let-7-5p candidate, miRNA3 (Fig. 3D and table S14). We further performed reciprocal miRNA pulldown-qPCR using probes against these three miRNAs (table S13). The miRNA1/2 probe mixture and the miRNA3 probe significantly enriched both *lncRNA1* and *pitx* transcripts compared with scrambled probes and with miRNA5, the most abundant background miRNA in this tissue (Fig. 3E and table S15). In situ hybridization further showed that these miRNAs are localized to the mantle fold region, overlapping with the *pitx-lncRNA1* expression domain (Fig. 3F). Together, these data support a reproducible association between miRNA1/2/3 and the *lncRNA1*-*pitx* transcripts.

TargetScan (*41*) detected no canonical seed-pairing sites between these miRNAs and *lncRNA1* or *pitx*, except for one weak 6-mer match between miRNA3 and *pitx*, arguing against a canonical seed-mediated targeting model (table S16). Instead, if these miRNAs participate in the *lncRNA1-pitx* module, they may do so through noncanonical or RNP-associated interactions, and their precise mechanistic role remains to be determined. In parallel, chromatin interaction analysis revealed left/right differences in local chromatin configuration around the *pitx-lncRNA1* locus. The most prominent feature was a strong loop in the right mantle between *lncRNA1* and a 707.5 kb downstream TAD boundary, whereas this interaction was absent in the left mantle (Fig. 3G). Because TAD boundaries can delimit regulatory domains and influence communication between regulatory elements and target genes (*42*, *43*), this right-specific interaction is consistent with a more constrained local regulatory configuration. These data suggest that chromatin organization is an important layer underlying the coordinated expression of *lncRNA1* and *pitx*.

Together, these observations suggest that the asymmetric *pitx-lncRNA1* locus is associated with side-specific chromatin organization, with the right-mantle *lncRNA1*-TAD-boundary loop representing the most prominent architectural difference. In addition, the overlapping cytoplasmic expression of *lncRNA1* and *pitx* is consistent with a post-transcriptional relationship affecting *pitx* mRNA abundance. miRNA1/2/3 are reproducibly associated with both transcripts and may participate in this cytoplasmic layer, but their precise mechanistic role remains unresolved.

### Developmental and evolutionary conservation of the *lncRNA1-pitx* association in gastropods

**Fig. 4.**
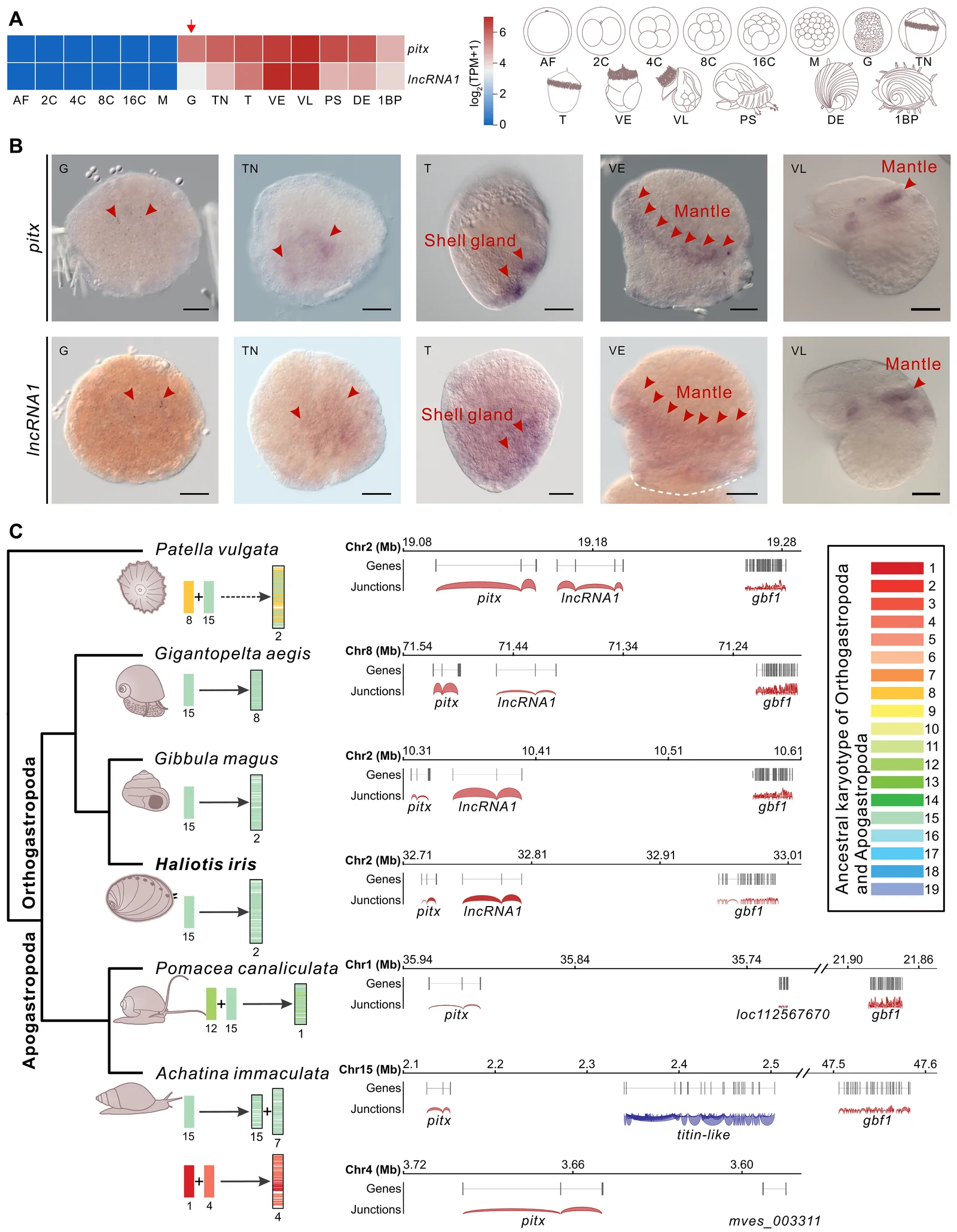
Developmental expression and conserved genomic association of *lncRNA1* and *pitx* in gastropods. (**A**) Expression dynamics of *pitx* and *lncRNA1* during *Haliotis discus* embryonic development; AF: artificial fertilization; 2C: 2-cell; 4C: 4-cell; 8C: 8-cell; 16C: 16-cell; M: morula; G: gastrula; TN: non-hatched trochophore; T: trochophore; VE: early veliger; VL: late veliger; PS: peristomial shell larva; DE: differentiation epipodes; 1BP: emergence of 1st breathing pore. (**B**) Whole-mount in situ hybridization showing the spatial expression of *pitx* and *lncRNA1* during representative embryonic and larval stages. Red arrowheads indicate major expression domains, including the shell gland and mantle region. (**C**) The conserved synteny between *pitx* and *lncRNA1* can be traced back to the ancestor of gastropods; their loci are consistently located on the ancestral chromosome 15 of Orthogastropoda and Apogastropoda and its descendants; blue junctions indicate that the genes are located on complementary strands.

Transcriptome analysis showed that both *lncRNA1* and *pitx* expression initiates at the gastrula stage (Fig. 4A), and they show overlapping expression domains throughout development, closely associated with the formation of the mantle and other organs (Fig. 4B). This suggests that the *lncRNA1-pitx* association is established early during abalone development and is spatially associated with mantle and organ formation. On a macroevolutionary scale, although ncRNAs are often difficult to compare across deep lineages (*44*, *45*), our synteny analysis revealed that the *lncRNA1* and *pitx* loci remain tightly linked throughout gastropod evolution (except in Apogastropoda) and are consistently located on the ancestral chromosome 15 of Orthogastropoda and Apogastropoda and their descendants (Fig. 4C and table S17). To test whether a conserved functional relationship accompanies this conserved genomic association, we performed cross-species validation in two experimentally accessible surrogate representatives from the corresponding major clades, trochid *Monodonta labio* and the limpet *Cellana toreuma* (fig. S9). In both species, *pitx* and *lncRNA1* showed concordant left-biased expression (fig. S9, A and D; table S18), consistent with the pattern observed in *H. iris*. RNAi assays further supported the same directional relationship as in *H. iris*: knockdown of *lncRNA1* reduced *pitx* expression, whereas knockdown of *pitx* did not significantly affect *lncRNA1* expression (fig. S9, B and E; table S19). In situ hybridization additionally revealed overlapping expression of the two transcripts in both species (fig. S9, C and F). Together, these results support a conserved *lncRNA1-pitx* regulatory association across the sampled gastropod lineages, consistent with deep conservation of this genomic association and suggesting that its functional relationship may also be evolutionarily conserved. In *H. iris*, our additional data further suggest that this association may include a miRNA-associated cytoplasmic component, although broader testing will be required to determine whether this specific molecular architecture is conserved across gastropods.

### Chitin-binding proteins are associated with melanin deposition

**Fig. 5.**
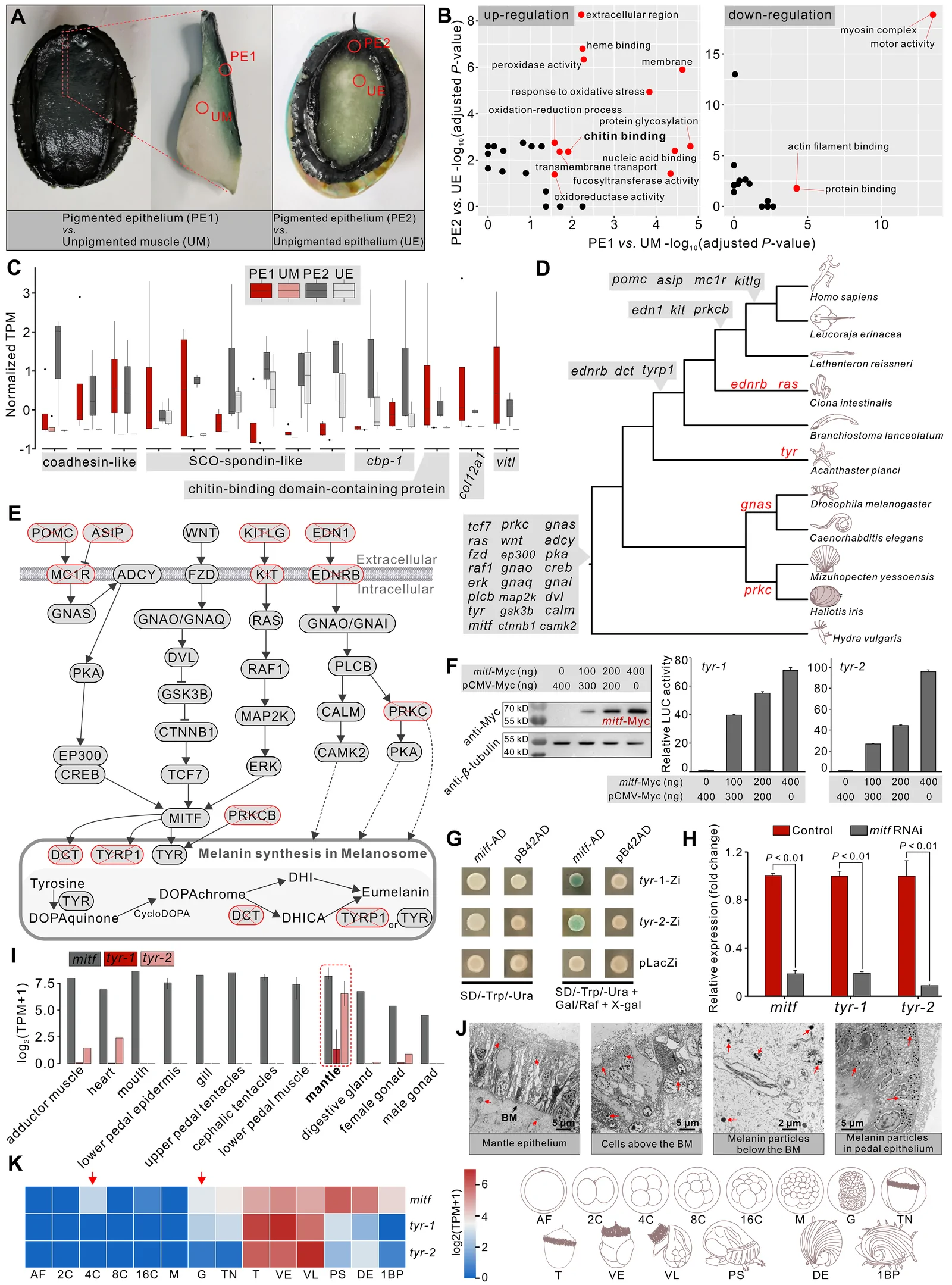
Melanin deposition, melanogenesis pathway evolution, and mantle-based melanin synthesis in *Haliotis iris*. (**A**) Illustration of normal and pigment-defective rainbow abalone variants and sampled tissues. The variant image is also used in fig. S10 to demonstrate the correlation between macroscopic tissue pigmentation and the microscopic presence of melanin. (**B**) GO enrichment of genes differentially expressed in pigmented *vs* unpigmented tissues; red dots indicate terms significant in both comparisons, black dots indicate terms only significant in one comparison. (**C**) Expression levels of chitin-binding related genes across four tissues. (**D**) Origins (gray background) and losses (red) of melanogenic genes across metazoan phylogeny. (**E**) Schematic of known eumelanin synthesis and regulation; genes lost in rainbow abalone are marked with red crosses. (**F**) Dual luciferase assays showing transcriptional regulation of *tyr* genes by rainbow abalone MITF; Western blots on the left show increased MITF expression with higher *mitf*-Myc vector concentrations. Data are mean ± SEM; *n* = 3 independent transfection experiments. (**G**) Yeast one-hybrid assays validating MITF regulation of *tyr*; green patches indicate positive results. (**H**) qRT-PCR results showing *mitf* knockdown reduces *tyr* expression. Data are mean ± SEM; *n* = 3 per group. (**I**) *mitf* and *tyr* expression across rainbow abalone tissues. (**J**) Transmission electron micrographs of abalone mantle and foot; red arrows indicate melanin particles, BM: basement membrane. Note that the second TEM panel in (J) is also presented in Fig. 6H to show *slc26a5* expression region, while the fourth TEM panel in (J) is reproduced in fig. S10 to facilitate comparison of melanin distribution between pigmented and unpigmented tissues. (K) Expression dynamics of *mitf* and *tyr* during *Haliotis discus* embryonic development; embryonic stages indicated by abbreviations refer to Fig. 4A. Significance was assessed using unpaired two-tailed Student’s *t* test for (F), and (H).

Melanin is a ubiquitous pigment in metazoans, responsible for the coloration of skin, hair, and eyes, and serving functions in photoprotection and immunity (*5*, *7*, *46*, *47*). While melanin synthesis in vertebrates proceeds via a well-characterized tyrosine metabolic pathway, the pathway in most other taxa remains poorly understood (*13*–*15*). The all-black skin of *H. iris* distinguishes it from other abalone species (Fig. 1A); however, some individuals exhibit an unpigmented lower pedal region (Fig. 5A). Electron microscopy confirmed a lack of melanin in this unpigmented tissue (fig. S10). This natural variation in melanin deposition offers an excellent model for investigating melanogenesis in gastropods. We performed comparative transcriptomics between pigmented and unpigmented tissues from both normal and variant individuals, comparing pigmented epithelium to unpigmented muscle and pigmented epithelium to unpigmented epithelium (Fig. 5A and fig. S11). Results showed 1,549 up- and 1,054 down-regulated genes in pigmented regions in either comparison (tables S20, S21). To focus on more robust pigmentation-associated signals, we prioritized genes showing concordant differential expression across both contrasts (182 up / 214 down). However, no known melanogenic genes were identified among them, and the key gene *tyr* was undetectable in any transcripts from these tissues (table S22). This suggests that melanin in the lower pedal is not synthesized in situ and that the color variant is likely determined by factors affecting melanin deposition rather than synthesis.

Transcriptome analyses revealed that chitin-binding genes, including *coadhesin-like* and *SCO-spondin-like*, were consistently upregulated in melanin-deposited tissues (Fig. 5, B and C). These genes were also enriched in GO terms associated with the cell membrane and extracellular region. Previous studies have found melanin deposited within cross-linked chitin structures in cuttlefish beaks, oyster shells, and silkworm epidermis (*48*–*51*). Based on these observations, we hypothesize that chitin-associated matrices may contribute to melanin deposition in *H. iris*. Consistent with this interpretation, additional histological and in situ analyses showed that pigmented epithelium is enriched not only for melanin, but also for chitin signal and for the expression of representative chitin-binding-related genes (fig. S12). Because these data remain correlative, they should be viewed as supporting evidence for a working hypothesis rather than direct proof of mechanism.

### Identification of the core melanogenesis pathway

Since transcriptomic comparisons of pigmented tissues were uninformative for identifying melanogenic genes, we used an evolutionary genomics approach to uncover the regulatory cascade. Melanogenesis mediated by *tyr* is evolutionarily conserved across metazoans (*46*, *47*, *52*), implying an ancient regulatory pathway. Melanin in *H. iris* is eumelanin, which is also *tyr*-driven in mollusks (*22*, *52*, *53*). We therefore investigated the origin and loss of all genes in the eumelanogenic pathway (ko04916) across metazoans (Fig. 5D). Of the three canonical eumelanogenic genes (*dct*, *tyrp1*, and *tyr*), only *tyr* exists in non-chordate metazoans. This suggests that the synthesis of melanin in *H. iris* is dominated by *tyr* and proceeds through the 5,6-dihydroxyindole (DHI) approach (Fig. 5E).

The *H. iris* genome contains two *tyr* paralogs (*tyr-1* and *tyr-2*). Among candidate upstream regulators, only the transcription factor *mitf* is conserved across non-chordate metazoans (Fig. 5, D and E). Dual-luciferase reporter assays in HEK293T cells showed that MITF positively regulated both *tyr* paralogs (Fig. 5F; figs. S13 and S14; table S23), and this interaction was further supported by yeast one-hybrid assays (Fig. 5G). Importantly, in vivo RNAi in live abalones showed that knockdown of *mitf* led to significant downregulation of both *tyr-1* and *tyr-2* (Fig. 5H and table S24). As an additional specificity control, several upstream or pathway-related genes (*ctnnb1*, *dvl*, *gnao*, *gsk3b*, *ras*, and *tcf7*) showed no significant expression change after *mitf* RNAi (fig. S15 and table S25), arguing against a general transcriptional suppression effect. Together, these results support MITF as a key positive regulator of *tyr* in *H. iris*. Regarding the upstream regulation of MITF, our comparative genomic survey showed that key components of alternative pathways are absent, whereas the core *wnt* signaling genes are broadly conserved across metazoan lineages (Fig. 5, D and E). We therefore interpret the *wnt-mitf-tyr* cascade as a plausible ancestral regulatory framework for melanogenesis, while noting that direct functional validation of the upstream *wnt-mitf* connection will require further study.

### The mantle is the primary site of melanogenesis

If melanin in the lower pedal is not synthesized locally, what is its origin? To identify the source tissue, we examined the expression patterns of the key melanogenic genes, *mitf* and *tyr*, across different organs. The two *tyr* paralogs were predominantly expressed in the mantle and were barely detectable in most other organs, including the lower pedal (Fig. 5I). Although *tyr-2* showed some expression in the heart, adductor muscle, and female gonad, there is no evidence that connective tissues link these organs to the lower pedal skin. This strongly implies that cutaneous melanin in *H. iris* is primarily produced in the mantle and subsequently transported. Electron microscopy confirmed the presence of noticeable melanin particles beneath the basement membrane of the mantle epithelium (Fig. 5J), validating the melanogenic function of the mantle.

Intriguingly, while the mantle is the primary site of *tyr* expression in adults (especially *tyr-1*, Fig. 5I), *tyr* expression begins at the gastrula stage (Fig. 5K), which precedes the formation of the mantle at the shell gland of the trochophore larva (*54*, *55*). This temporal discrepancy may be attributed to either the pleiotropy of *tyr* or a deeper, yet unknown, link between *tyr* and mantle development, such as cues that the mantle forms earlier.

### *Prestin* genes suggest a potential mechanosensory specialization of the mantle

**Fig. 6.**
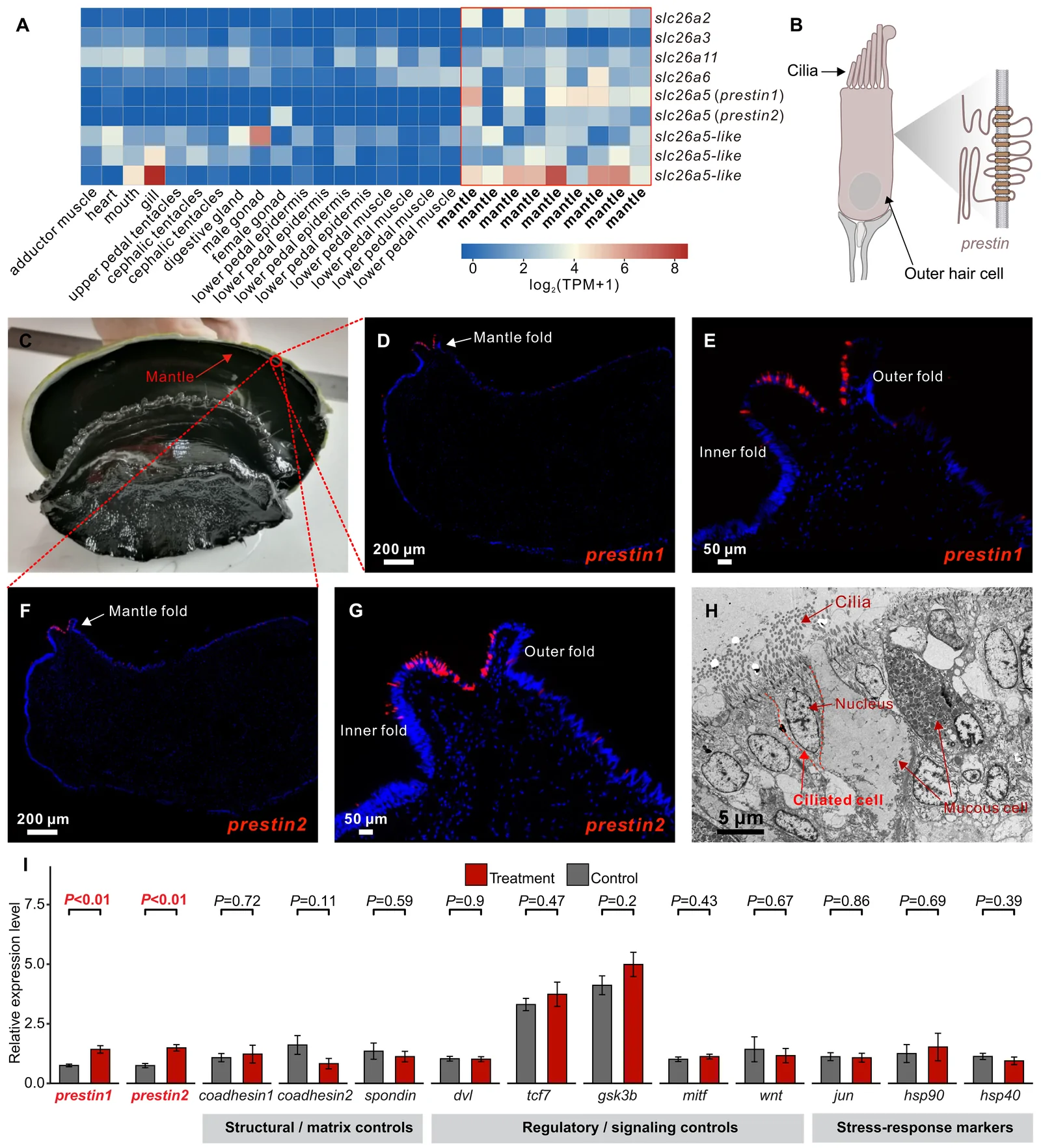
Specific expression and vibration responsiveness of *slc26a5* genes in the rainbow abalone mantle. (**A**) Expression levels of solute carrier family 26 members across tissues. (**B**) Schematic of outer hair cell structure and *prestin* (mammalian *slc26a5*) localization in mammals. (**C**) Photo of the rainbow abalone mantle. This image is the same as the example shown in Fig. 1A, reproduced here to indicate the localization of the mantle. (**D** to **G**) In situ localization of two *slc26a5* paralogs in the rainbow abalone mantle. (**H**) Transmission electron micrograph of the *slc26a5* expression region in the rainbow abalone mantle. This image is the same as the second panel in Fig. 5J, reproduced here to show the *slc26a5* expression region. (**I**) qRT-PCR analysis of *prestin* and control gene expression in mantle tissue after simulated water-flow vibration treatment. *prestin1* and *prestin2* were significantly upregulated after vibration treatment, whereas mantle structural/matrix-associated genes, representative regulatory/signaling genes, and stress-response marker genes showed no significant induction. Data are mean ± SEM; *n* = 8 per group. Significance was assessed using independent-samples *t* tests.

In addition to its established role in shell formation (*56*) and melanogenesis, the mantle may also contain a previously underappreciated sensory specialization. This possibility is suggested by the specific and high expression of multiple members of the solute carrier family 26 (*57*) in mantle tissue (Fig. 6A). Among them, *slc26a5* (*prestin*) is of particular interest because its vertebrate and insect homologs are associated with sensory epithelial function (Fig. 6B) (*58*–*61*), and one *H. iris* paralog (*prestin1*) retains a transmembrane architecture highly similar to that of mammalian *prestin* (table S26) (*62*). In *H. iris*, the two *slc26a5* genes are expressed almost exclusively in the mantle and are specifically localized to the inner and outer mantle folds, which are rich in ciliated cells (Fig. 6, C to H).

To further test whether these genes are associated with sensory responsiveness, we performed a simulated water-flow vibration experiment. After controlled vibration treatment, both *prestin1* and *prestin2* were significantly upregulated in mantle tissue, whereas additional mantle structural/matrix genes, representative signaling genes, and stress-response markers did not show significant induction (Fig. 6I and table S27). These results indicate that the mantle *prestin* genes are vibration-responsive and are consistent with a possible role in sensing or responding to hydrodynamic disturbance. Further functional work will be required to determine whether this system mediates true mechanosensory transduction in abalone.

Taken together, these results establish a chromosome-level genomic framework for *H. iris* and link conserved genome organization with three focal biological themes: mantle asymmetry, melanogenesis, and mantle multifunctionality. Our analyses show that *H. iris* retains a relatively conserved genomic architecture, including a stable karyotype and ancestral *Hox* and *ParaHox* gene configurations, supporting its value as a model for understanding deep gastropod evolution. A central finding is the identification of a multi-layered regulatory association linked to mantle asymmetry. Extending beyond the established role of *pitx*, we identify a syntenically conserved *lncRNA1* whose knockdown reduces *pitx* expression. In *H. iris*, this association is supported by chromatin-contact evidence and exploratory data consistent with a possible miRNA-associated post-transcriptional contribution, while comparative analyses support broader conservation of the *lncRNA1*-*pitx* association across gastropods.

Our study also provides molecular insight into gastropod mantle multifunctionality. We support *mitf* as a key positive regulator of *tyr*, show that the mantle is the primary site of melanin synthesis, and identify a vibration-responsive prestin-like module consistent with a possible sensory specialization. Together, these genomic and functional analyses refine the molecular understanding of asymmetry, pigmentation, and mantle multifunctionality in the rainbow abalone and provide a useful resource for future studies of molluscan development and evolution.

## MATERIALS AND METHODS

### Sample collection and sequencing

Rainbow abalone *H. iris* samples were obtained commercially and acclimated in seawater at 18°C for two days. Subsequently, the following tissues were dissected and flash-frozen in liquid nitrogen (table S1): upper pedal muscle (unpigmented), lower pedal epithelium (pigmented), lower pedal epithelium (unpigmented), lower pedal muscle (unpigmented), left and right mantle lobes, adductor muscle, gill, mantle, digestive gland, upper pedal tentacles, mouth, heart, male gonad, female gonad, and left and right cephalic tentacles. These samples were used for extracting genomic DNA, sequencing library construction (Oxford Nanopore long reads and Illumina 150 bp paired-end reads), transcriptomics, Hi-C, and methylome. Oxford Nanopore sequencing was performed on the PromethION platform and other libraries were sequenced on the Illumina NovaSeq 6000 platform. All sequencing was conducted at Novogene (Tianjin, China).

### Genome assembly

Illumina NGS reads were quality filtered using fastp v0.23.1 (*63*) (default parameters) and then subjected to 23-mer frequency analysis using Jellyfish v2.2.10 (*64*). GenomeScope 2.0 (*65*) was used to estimate the genome size based on the *k*-mer distribution.

Nanopore reads were quality controlled with Ontbc v1.1 (parameters: read length > 1,000 bp, quality score > 8; https://github.com/FlyPythons/ontbc) and then assembled into contigs using NextDenovo v2.4.0 with default parameters (https://github.com/Nextomics/NextDenovo). The preliminary assembly was polished in two rounds using the filtered Nanopore reads and then in three rounds using the Illumina reads with NextPolish (parameters: -min_read_len 5 k -max_depth 100; https://github.com/Nextomics/NextPolish).

The Hi-C reads were aligned to the polished genome using Juicer v1.5 (*66*), and a Hi-C contact matrix was generated. The 3D-DNA v180922 (*67*) was then used to scaffold the genome into chromosome-level *pseudo*-haplotypes with default parameters. The assembly was further curated in Juicebox (*68*) to obtain the final version.

Genome completeness was assessed by benchmarking universal metazoan genes (metazoa_odb10) using BUSCO v4 (*69*). Illumina reads were aligned to the final assembly using BWA v0.7.15-r1140 (*70*). After removing ambiguous mappings, the single-base error rate was estimated using an in-house perl script.

### Transcriptome assembly and annotation

RNA-seq reads from all tissues were filtered with fastp v0.23.1 (*63*) and then assembled into transcripts using Trinity v2.8.5 (*71*). Open reading frames (ORF) were identified from the transcripts using TransDecoder v5.5.0 (https://github.com/TransDecoder/TransDecoder) with default parameters. Only transcripts with complete ORFs were retained for further analysis.

### Genome annotation

Repetitive elements were annotated using both *de novo* and homology-based approaches. RepeatMasker v2.1 (*72*) and the RepeatProteinMask module were used to identify known repeats based on Repbase. RepeatModeler v1.0.11 (*73*) (parameters: -species Haliotis -nolow -norna -no_is) and LTR_FINDER (*74*) generated a *de novo* repeat library, which was used by RepeatMasker v2.1 (*72*) to find additional repeats. Tandem repeats were identified by Tandem Repeat Finder v4.07 (*75*) with the following parameters: “Match = 2, Mismatch = 7, Delta = 7, PM = 80, PI = 10, Minscore = 50”.

For protein-coding genes, evidence from three approaches was integrated: (1) *de novo* prediction with AUGUSTUS v3.2.1 (*76*) using the “seahare” model; (2) Homology-based prediction by aligning proteins from 8 molluscan genomes, *Aplysia californica* (GCA_000002075.2), *Biomphalaria glabrata* (GCA_947242115.1), *Crassostrea gigas* (GCA_011032805.1), *Lottia gigantea* (GCA_000327385.1), *Octopus bimaculoides* (GCA_001194135.2), *Pecten maximus* (GCA_902652985.1), *P. canaliculata* (GCF_003073045.1), and *Pomacea maculata* (GCA_003073045.1), to the genome using tBLASTN v2.7.1 (*77*) (e-value 1e-5) and defining gene models with GeneWise (*78*); (3) RNA-seq support by aligning transcripts to the genome using BLAT v35 (*79*) and PASA (*80*) to generate gene models. The three lines of evidence were combined using EvidenceModeler v1.1.1 (*81*) to obtain a non-redundant gene set. Functional annotation was performed by scanning the gene set against databases including InterPro, GO, KEGG, UniProt, COG, Pfam, and NR using InterProScan v5.30–69.0 (*82*).

### Phylogenetics and demographic history

High-quality genomes of 14 mollusks were retrieved from NCBI, including *Haliotis rufescens* (GCA_023055435.1), *Haliotis laevigata* (GCA_008038995.1), *Haliotis rubra* (GCA_003918875.1), *Lanistes nyassanus* (GCA_004794575.1), *Marisa cornuarietis* (GCA_004794655.1), *P. canaliculata* (GCF_003073045.1), *Pomacea maculata* (GCA_004794325.1), *B. glabrata* (GCA_947242115.1), *Achatina immaculata* (GCA_009760885.1), *Achatina fulica* (http://gigadb.org/dataset/100647), *Cepaea nemoralis* (GCA_014155875.1), *A. californica* (GCA_000002075.2), *L. gigantea* (GCA_000327385.1), and *Mizuhopecten yessoensis* (GCA_002113885.2). Orthologous genes were identified by performing ALL-to-ALL reciprocal BLAST searches using Diamond v2.0.4 (*83*). Individual gene trees and a supermatrix tree were constructed using IQ-TREE v1.6.12 (*84*) after alignment with MUSCLE v3.8.425 (*85*) and PAL2NAL v14 (*86*). A coalescent-based species tree was generated with ASTRAL v5.5.9 (*87*).

Divergence times were estimated using MCMCtree in PAML v4.9 (*88*), with calibrations from fossils and TimeTree (*89*, *90*): the most recent common ancestor (MRCA) of *Lanistes nyassanus* and *P. canaliculata* > 150 Mya; MRCA of *L. gigantea* and *A. californica*: 470.2–531.5 Ma; MRCA of *H. iris* and *H. rubra*: >84.9 Ma; MRCA of *B. glabrata* and *A. fulica*: 177–187 Ma; MRCA of *A. californica* and *Achatina immaculata*: 91–430 Ma; MRCA of *P. canaliculata* and *Achatina immaculata*: 334–489 Ma; MRCA of *H. iris* and *Achatina immaculata*: 401–507 Ma; MRCA of *Mizuhopecten yessoensis* and *Achatina immaculata*: 520–568 Ma.

The genome-wide substitution rates for each lineage were estimated using R8s v1.8.1 (*91*) with penalized likelihood and the same dataset and calibration points as above. For demographic history, SNPs were identified from aligned Illumina reads using SAMtools (*92*) (parameters: mpileup -q 20 -Q 20). Demographic changes were reconstructed using PSMC v0.6.5 (*93*) with a generation time of 4 years (*94*).

### Chromosome evolution analysis

Six gastropod species were selected: *P. vulgata* (GCF_932274485.2), *Gigantopelta aegis* (GCF_016097555.1), *G. magus* (GCA_936450465.1), *H. iris* (this study), *Achatina immaculata* (GCA_009760885.1), and *P. canaliculata* (GCF_003073045.1). Syntenic blocks between chromosomes were identified using MCScanX v1.1 (*95*) (parameters: “-s 3 -w 25 -b 2 -m 100 -k 200 -g -0.2”) given the low conservation between molluscan genomes. Ancestral karyotypes were constructed using ANGeS v1.01 (*96*) based on synteny to examine chromosomal evolution across lineages. The syntenic blocks inferred from this analysis to represent ancestral proto-chromosomal fragments were then further projected onto the chromosomes of 12 additional gastropod species (table S28) through reciprocal best hit (RBH)-based homology mapping, allowing a broader assessment of chromosomal evolutionary patterns across gastropods.

### Gene family analysis

Protein sequences from the 15 mollusks above were clustered using OrthoFinder v2.3.1 (*97*). Expansions and contractions of orthogroups were analyzed using CAFÉ v4.0.1 (*98*) with default parameters. Significance was determined based on both family-wide and Viterbi *P*-values <0.01 (*99*).

### Identification of homeobox and melanogenesis genes

The protein sequences of 11 *Hox* genes and three *ParaHox* genes were obtained according to the annotations reported by Wang *et al.* (*34*) and used for comparative analysis. These sequences were aligned to each genome using BLAT v.35 (*79*) to identify all candidate hits, retaining matches over 50% of the query length. Targeted sequences plus 10 kb up- and downstream were extracted and genes were precisely predicted using Genewise v2.4 (*78*). Redundant sequences were removed to obtain a non-redundant homeobox gene entry, which was verified by searching against the NR database.

Genomes from 10 representative metazoan genomes, *Homo sapiens* (GCA_000001405.29), *Leucoraja erinacea* (GCA_028641065.1), *Lethenteron reissneri* (GCA_015708825.1), *Ciona intestinalis* (GCA_009617815.1), *Branchiostoma lanceolatum* (GCA_927797965.1), *Acanthaster planci* (GCA_001949145.1), *Drosophila melanogaster* (GCA_000001215.4), *Caenorhabditis elegans* (GCA_000002985.3), *Mizuhopecten yessoensis* (GCA_002113885.2), and *Hydra vulgaris* (GCA_022113875.1), were combined with that of *H. iris* to generate a simplified metazoan phylogeny. The emergence and loss of melanogenic genes across this phylogeny were assessed using the same approach as described above. Key melanogenic genes were identified mainly based on the KEGG pathway ko04916.

### Topologically associating domain (TAD) analysis and loop calling

Hi-C sequencing data representing spatial interactions between loci were used to examine *pitx* region contacts. Resolution was first assessed using HiC-Pro v3.1.0 (*100*) with parameters “BIN_SIZE = 5000 10000 15000 20000 25000 30000 35000 40000 100000 200000” to evaluate contact matrices and determine the highest resolution where >80% of bins show >1000 contacts.

TADs were then identified from this optimal resolution matrix using “hicFindTADs” in HiCExplorer v1.5.12 (*101*), which measures separation between adjacent regions, with parameters “--minDepth 30000 --maxDepth 100000 --step 10000 --thresholdComparisons 0.05 --delta 0.05 --correctForMultipleTesting fdr”. TADs were defined as local minima in these TAD separation scores. TADs were visualized using pyGenomeTracks v3.7 (*102*).

Hi-C loop calling was conducted using “hicDetectLoops” in HiCExplorer v1.5.12 (*101*) with parameters: “--maxLoopDistance 2000000 --windowSize 10 --peakWidth 6 --pValuePreselection 0.05 --pValue 0.05”.

### Transcriptome differential expression analysis

RNA-seq reads from each *H. iris* tissue were filtered using fastp v0.23.1 (*63*) (parameters: -n 0), then aligned to the genome using Hisat2 v2.1.0 (*103*). StringTie v2.2.1 (*104*) was used to calculate the read counts of each gene and quantify gene expression as TPM values based on the gene annotations GFF. Differentially expressed genes were identified using DESeq2 (*105*), requiring |log_2_FC| ≥ 1 and FDR < 0.05. GO enrichment analysis was performed to examine functions of differentially expressed genes (Chi.FDR < 0.05).

To identify the candidate differentially expressed non-coding elements in left and right mantles, transcripts obtained from mantles without ORFs were aligned to the genome using PASA (*80*) to define structures. These were filtered against protein-coding annotations, resulting in 95,395 non-coding elements active in the mantle (table S4). The paired-end oligo-dT libraries were used to capture the strand information (table S1). Finally, the annotation file of these non-coding elements (GFF) was used to quantify and identify their differentially expressed genes as described above for coding genes.

### DNA methylation analysis

Paired-end whole-genome bisulfite sequencing reads were filtered using fastp v0.23.1 (*63*) with default parameters. The *H. iris* genome was converted using “bismark_genome_preparation” in Bismark v0.23.0 (*106*), which performs in silico C- > T and G- > A conversions. Filtered reads were mapped to the converted genome using Bowtie2 v2.3.4.3 (*107*). Methylation information at all C positions was extracted using “bismark_methylation_extractor” in Bismark v0.23.0 (*106*) to obtain data for CpG/CHG/CHH contexts. Differentially methylated positions were identified using the R package “*methylKit*” (*108*) requiring at least 20% methylation change and FDR < 0.05.

### Quantitative analysis of genes

The mRNA expression levels of selected genes were quantified by quantitative reverse transcription PCR (qRT-PCR) using a QuantStudio thermal cycler (Applied Biosystems, USA) and SYBR Green 2× Master Mix (Takara, Japan). The analyzed genes included *pitx*, *lncRNA1*, *lncRNA2*, *tyr-1*, *tyr-2*, *mitf*, *prestin1* and *prestin2* (two *slc26a5* paralogs), as well as additional genes examined in the MITF-related pathway, RNAi specificity controls, and vibration-response controls. Gene-specific primers are listed in table S29. Total RNA was reverse-transcribed into cDNA, which was then used as the template for qRT-PCR amplification. Relative expression levels were normalized using one of the reference genes (*GAPDH*, *EF-1a*, *RS18* or β*-actin*), depending on the corresponding experiment and sample set. PCR cycling conditions were as follows: 95°C for 30 s, followed by 40 cycles of 95°C for 5 s and 60°C for 30 s. Melting-curve analysis was performed after each run to confirm amplification specificity. Relative transcript abundance was calculated using the 2^-ΔΔCt^ method.

For the reciprocal RNAi experiments, expression changes of *pitx*, *lncRNA1*, and *lncRNA2* were quantified after knockdown of each gene. For the melanogenesis-related experiments, expression of *tyr-1*, *tyr-2*, and selected upstream or pathway-related genes was measured following *mitf* RNAi. For the vibration-response assay, expression of *prestin1*, *prestin2*, and selected mantle-expressed control genes was quantified in stimulated and control animals. Statistical analyses were performed using unpaired two-tailed Student’s *t* tests for two-group comparisons (except for left-right paired comparisons, which were analyzed using paired samples *t* tests) and one-way ANOVA for multiple-group comparisons. Differences were considered significant at *P* < 0.05 unless otherwise stated.

### RNA interference

For each target gene, three non-overlapping siRNAs targeting different regions of the target transcript were designed and pooled for injection. A scrambled/negative-control (NC) treatment was included in parallel. Experimental animals were allocated to RNAi and NC groups (typically *n* = 3–9 per group). Each individual received a first injection of 20–50 μg pooled siRNA dissolved in PBS into the adductor muscle, followed by a second injection of the same amount 12 h later to improve knockdown efficiency. Mantle tissues were collected 12 h after the second injection for RNA extraction and qRT-PCR analysis. Because siRNAs were delivered into the adductor muscle whereas expression was assayed in mantle tissue, the effective concentration reaching mantle cells is expected to be substantially lower than the nominal injected concentration. Statistical significance of differences between RNAi and NC groups was determined using unpaired two-tailed Student’s *t* tests. All siRNA and qRT-PCR primer sequences are detailed in the table S29.

### Histological staining and in situ hybridization

For the histological survey, we employed conventional paraffin sectioning techniques to prepare histological sections of the left anterior mantle lobe in the rainbow abalone. The preparation of sections and hematoxylin-eosin (HE) staining followed previously published protocols (*109*) and were meticulously optimized based on the preservation conditions of the samples (e.g., sample fixation, section thickness, etc.).

Fluorescent in situ hybridization was performed and optimized according to the method described by Kishi *et al.* (*110*). Briefly, after rehydration, digestion, and pre-hybridization (37°C for 1 h), left mantle tissue sections were hybridized with hybridization probes (500 nM, designed and synthesized by commercial providers; table S13) at 40°C overnight. The probes were then washed away with pre-warmed 2 × SSC, 1 × SSC, and 0.5 × SSC. After extensive washing, sections were added branch hybridization solution and held at 40°C for 45 min in a humid chamber. The sections were then washed with 2 × SSC and rinsed in 1 × PBS. Subsequently, the sections were incubated in fluorescent hybridization solution (1:400 iF488-Tyramide, Cy3, or Cy5 dye) and incubated at 42°C for 3 hours. After washing consecutively with 1 × PBS, the sections were counterstained with DAPI reagent. Finally, sections were visualized under a fluorescence microscope (NIKON, ECLIPSE CI, Japan), and images were captured. Fluorescence signals were acquired using appropriate filter configurations: blue fluorescence (typically DAPI for nuclear counterstain, UV excitation 330–380 nm, emission ∼420 nm), green fluorescence (generated via iF488-Tyramide signal amplification, excitation ∼470–495 nm, emission ∼510–530 nm), red fluorescence (CY3 conjugated probe, excitation ∼510–560 nm, emission ∼590 nm), and yellow fluorescence (CY5 conjugated probe, excitation ∼656 nm, emission ∼670 nm).

Whole-mount in situ hybridization (WISH): The probes of *lncRNA1* and *pitx* were synthesized using specified primers (table S29) from the cDNA library of the left lobe of the mantle. Plasmid-derived riboprobes were synthesized using T7 polymerases, tagged with digoxigenin labels (*111*). Concurrently, abalone embryos at various developmental stages, including artificial fertilization, 2-cell stage, 4-cell stage, 16-cell stage, morula, gastrula, non-hatched trochophore, trochophore, early veliger, and late veliger stages, were sequentially collected and rinsed with RNase-free water. The embryos underwent thorough washes with a dedicated solution and DEPC-treated water, followed by Proteinase K digestion. Subsequently, they were washed with 0.1 mol/L glycine solution, PBS, and 5 × SSC solution. The hybridization and immunological detection procedures were carried out as previously described (*34*, *112*).

### *lncRNA1*-miRNAs pulldown and miRNA annotation

To identify miRNAs interacting with the target *lncRNA1* in abalone, HyPro-proximity miRNA pulldown (*40*) was performed by Aksomics (Shanghai, China). Briefly, tissues were ground in liquid nitrogen, and a portion was reserved as the input control. For the experimental group, samples underwent DSP crosslinking, ethanol permeabilization, and hybridization with a custom DIG-conjugated DNA probe specific to the target *lncRNA1* (table S13). The HyPro fusion enzyme (APEX2-DIG binding protein) was then incubated, followed by proximity-dependent biotinylation of adjacent RNA molecules using biotin-phenol and H_2_O_2_. After cell lysis, total RNA was extracted, and biotinylated RNAs were captured using streptavidin magnetic beads, followed by RNA elution and purification. Small RNA libraries were constructed from the enriched RNA fractions and the control RNA using the NEB Multiplex Small RNA Library Prep Set for Illumina (New England Biolabs, USA), involving 3′ and 5′ adapter ligation, cDNA synthesis, PCR amplification, and size selection of 135–155 bp PCR products (corresponding to 15–35 nt small RNAs). Library quality was assessed using an Agilent 2100 Bioanalyzer. Sequencing was performed on an Illumina platform using the TruSeq Rapid SR Cluster Kit. Raw sequencing reads were processed with Cutadapt (*113*) to remove adapters, and tags ≥17 nt were retained as trimmed reads for downstream analysis.

Following quality control, the trimmed reads were mapped to the *H. iris* genome using Bowtie2 (*107*) with the “--very-sensitive-local” preset parameter. Alignments with a mapping quality score greater than 20 were retained for further analysis. The SAMtools (*92*) and Bedtools (*114*) software suites were subsequently employed to derive genomic coordinates and quantify read counts for the successfully mapped reads. Captured candidate miRNA sequences were annotated by homology searches against the *H. rufescens* reference miRNA sequences in MirGeneDB 3.0 (https://www.mirgenedb.org/). BLASTN was performed using the short-read mode with the following parameters: -task blastn-short -reward 1 -penalty −2 -gapopen 0 -gapextend 2 -evalue 5. From the BLASTN output, only reads showing 100% identity and full-length alignment to the corresponding primary miRNA (pri-miRNA) or precursor miRNA (pre-miRNA) reference sequences were retained for annotation. Mature arm assignment, including 5p or 3p annotation, was then determined based on the best-matching reference sequence. The three mature miRNA sequences were further analyzed using TargetScan (*41*) with default parameters to identify potential canonical seed-matched target sites, including 8mer, 7mer-m8, 7mer-A1, and 6mer sites, in *pitx* and *lncRNA1*.

For quantification, to minimize bias caused by terminal sequence heterogeneity of isomiRs, reads that fully matched the reference genomic coordinates were used as anchors. All overlapping reads with minor length variations at the upstream or downstream ends within the anchored interval were merged and assigned to the corresponding miRNA locus. In contrast, unannotated reads were retained with their original mapping coordinates to preserve the spatial distribution of potential novel small RNA loci.

### Reciprocal miRNA pulldown-qRT-PCR

RNA pull-down was performed using the RNA Pull-Down Kit (Sangon Biotech, China) according to the manufacturer’s instructions with minor modifications. Briefly, the left mantle lobes from three abalones were dissected, minced, and crosslinked with 0.3% formaldehyde at room temperature for 10 min with gentle shaking. The crosslinking reaction was quenched by adding glycine to a final concentration of 125 mM and incubating for 5 min at room temperature. After washing with 1 × PBS, the tissues were lysed using lysis buffer supplemented with 2 μL RNase inhibitor (Accurate Biology, China). The lysate was then incubated with 4 μg biotinylated probes (a 1:1 mixture of random probe1 and random probe 2 for control group 1; the probe of miRNA5 for control group 2; a 1:1 mixture of miRNA1 probe and miRNA2 probe for experimental group 1; the miRNA3 probe for experimental group 2) at 4°C for 4 h with rotation. Meanwhile, streptavidin magnetic beads were washed with Wash I and resuspended in 1 × RNA binding buffer. The probe-lysate mixture was subsequently incubated with the prepared beads at 4°C for 3 h to capture probe-bound RNA complexes. After incubation, the beads were sequentially washed once with Wash I and three times with Wash II. Finally, 2 μg of exogenous RNA (total RNA of *Strongylocentrotus intermedius*) was added to the beads, and bound RNA was eluted and extracted for qRT-PCR analysis. Recovered *pitx* and *lncRNA1* transcripts were quantified by qRT-PCR. Relative RNA enrichment in each pulldown fraction was calculated using the 2^-ΔΔCt^ method after normalization to the exogenous RNA spike-in (*RS18* as the reference gene), with the random-probe pulldown used as the calibrator. Probe and primer sequences are provided in tables S13 and S29.

### Dual-luciferase reporter assays

Because molluscan cell lines suitable for routine transfection are not currently available, human HEK293T cells were used as a heterologous system for luciferase reporter assays. Cells were cultured in 24-well plates using high-glucose Dulbecco’s modified Eagle’s medium (HyClone, USA) supplemented with 10% FBS (Gibco, USA) and 1× penicillin/streptomycin (Solarbio, China) at 37°C with 5% CO_2_ and passaged every 2–3 days.

To evaluate the regulatory activity of abalone MITF on target gene promoters, reporter and expression plasmids were constructed. Promoter regions of rainbow abalone *tyr-1* or *tyr-2* genes were amplified by PCR from genomic DNA using KOD One PCR Master Mix (TOYOBO, Japan) with specific primers (table S29) designed based on gene sequences and predicted protein domains. Purified PCR products (DNA Gel Extraction Kit, Sangon Biotech) were inserted into the linearized pGL6-TA reporter plasmid (Beyotime, China) via In-Fusion cloning. Concurrently, an expression plasmid encoding abalone MITF was generated by cloning the *mitf* coding sequence into the pCMV-Myc expression vector (Clontech, Mountain View, USA) using In-Fusion cloning. For reporter assays, HEK293T cells seeded in 24-well plates were transfected at 60–70% confluency. Transfections included the appropriate promoter-reporter plasmid, the pRL-TK *Renilla* luciferase internal control plasmid (Promega), and either the MITF expression plasmid or an empty pCMV-Myc vector as a control, using Lipofectamine 3000 (Thermo Fisher Scientific, Waltham, USA). Cells were harvested 24–36 hours post-transfection. Firefly and *Renilla* luciferase activities were quantified using the Dual-Glo Luciferase Assay System (Promega). Firefly luciferase activity was normalized to *Renilla* luciferase activity and expressed as fold induction relative to cells transfected with the empty vector control. All promoter activity experiments were performed in triplicate.

### Abalone embryonic transcriptome

Due to the inaccessibility of *H. iris* embryos, *H. discus* embryos representing 14 developmental stages were used as a substitute: artificial fertilization, 2-cell, 4-cell, 8-cell, 16-cell, morula, gastrula, non-hatched trochophore, trochophore, early veliger, late veliger, peristomial shell larva, differentiation epipodes, and emergence of the 1st breathing pore. RNA was extracted from whole flash-frozen embryos for RNA-seq. Data was analyzed similarly to the tissue transcriptomes above.

### Synteny analysis of *pitx* and *lncRNA1*

To investigate the conservation and structural context of *pitx* and *lncRNA1*, RNA-seq reads for species pertinent to the chromosome evolution analysis (table S30) were retrieved from the NCBI database. Raw reads were subjected to quality filtering using fastp v0.23.1 (*63*) with the parameter “-n 0”. The filtered reads were then mapped to their respective reference genomes using HISAT2 v2.1.0 (*103*). Initial expression levels of the *pitx* gene were assessed using StringTie v2.2.1 (*104*). For RNA-seq reads exhibiting *pitx* expression, they were further processed with StringTie v2.2.1 (*104*) for reference-based transcript assembly. The resulting transcripts were subsequently refined using PASA (*80*) to identify accurate gene structures. Finally, the syntenic relationship between *pitx* and *lncRNA1* across these species was visualized using the IGV browser, based on the gene structure files (GFF, from PASA) and alignment files (bam, from HISAT2) generated from the aforementioned analyses.

### Cross-species functional validation of *pitx-lncRNA1* regulatory association

For cross-species functional validation of the *pitx-lncRNA1* regulatory relationship, two additional gastropod species were collected from Chinese coastal localities: the limpet *Cellana toreuma* from Shuangyue Bay, Huizhou, Guangdong Province, China, and the trochid *Monodonta labio* from Tieshangang, Beihai, Guangxi Province, China. These species were selected as experimentally accessible representatives of Patellogastropoda and Vetigastropoda, respectively, because the genome-referenced species identified in the comparative synteny analysis (*P. vulgata* and *G. magus*) were not available for live sampling. Fresh mantle tissues were dissected immediately after collection and used for qRT-PCR, RNA interference, and in situ hybridization analyses.

To test whether the *pitx-lncRNA1* regulatory relationship is conserved in additional gastropod lineages, homologous *pitx* and *lncRNA1* sequences were identified in *C. toreuma* and *M. labio*, and species-specific qRT-PCR primers and siRNAs were designed accordingly. For each species, left and right mantle tissues were first analyzed by qRT-PCR to examine expression asymmetry. RNAi experiments were then performed using gene-specific siRNAs targeting *lncRNA1* or *pitx*, together with the corresponding negative controls, following the same general strategy used in *H. iris*. After treatment, mantle tissues were collected for expression analysis. Relative transcript levels were quantified by qRT-PCR and calculated using the 2^-ΔΔCt^ method with internal reference genes. Statistical significance was generally evaluated using unpaired two-tailed Student’s *t* tests, except for left-right paired comparisons, which were analyzed using paired samples *t* tests. Primer and siRNA sequences are provided in table S29.

In situ hybridization was additionally performed on mantle tissues of *C. toreuma* and *M. labio* using species-specific probes targeting *pitx* and *lncRNA1* (table S29). Tissue fixation, sectioning, hybridization, washing, and imaging followed the optimized protocol used for *H. iris*, with minor adjustments according to tissue condition and section thickness. Co-localization of the two transcripts was evaluated in mantle sections from both species.

### Yeast one-hybrid assays

To validate MITF regulation of *tyr* genes, yeast one-hybrid assays were performed. Promoter regions of *tyr-1* and *tyr-2* containing predicted MITF binding sites were PCR amplified (table S29) and cloned into the pLacZi vector. *Mitf* coding sequences were cloned into the pB42AD vector (table S29). Plasmids were co-transformed into the yeast strain EGY48 and transformants selected on SD/−Trp/-Ura plates. Positive colonies were screened for reporter activation on chromogenic medium with X-gal.

### Transmission electron microscopy

To observe melanin particles in the abalone mantle, the ultrastructure was examined by transmission electron microscopy. Mantle tissue pieces (< 1 mm^3^) from fresh abalone were fixed in 2.5% glutaraldehyde. After rinsing with 0.1 M phosphate buffer, gradient acetone dehydration was performed. Tissue pieces were resin-embedded and cured at 37°C, 45°C, and 60°C sequentially. Ultrathin 70 nm sections were cut using a Reichert-Jung ultracut E ultramicrotome (Austria) and collected on copper mesh. Sections were stained with uranyl acetate and lead citrate before imaging on a JEM1200 electron microscope.

### Simulated water-flow vibration experiment

To investigate whether mantle-enriched *slc26a5* genes respond to mechanical / hydrodynamic stimulation, a simulated water-flow vibration experiment was performed using a vibration speaker as a surface transducer. Sixteen abalones were acclimated in seawater at 18°C for two days and then randomly assigned to control and experimental groups. In the experimental group, a vibration speaker was attached to the wall of the glass culture tank and driven by a frequency generator. Abalones were subjected to vibration stimulation at 120 Hz and 80 dB for 20 s followed by 10 s rest, repeated cyclically for 3 h. Control animals were maintained under the same conditions without vibration treatment. Mantle tissues were then dissected from both groups, and total RNA was extracted for qRT-PCR analysis. Expression levels of *prestin1* and *prestin2* were quantified, together with selected mantle-expressed control genes without known mechanosensory association. Relative transcript abundance was calculated using the 2^-ΔΔCt^ method with internal reference genes. Primer sequences are provided in table S29.

## References

[1] P. Bouchet, J. Frýda, B. Hausdorf, W. Ponder, A. Valdés, A. Warén, Classification and nomenclator of gastropod families. Malacologia 47, 1–397 (2005).

[2] K. M. Kocot, J. T. Cannon, C. Todt, M. R. Citarella, A. B. Kohn, A. Meyer, S. R. Santos, C. Schander, L. L. Moroz, B. Lieb, Phylogenomics reveals deep molluscan relationships. Nature 477, 452–456 (2011). 21892190 10.1038/nature10382 PMC4024475

[3] L. R. Page, Modern insights on gastropod development: Reevaluation of the evolution of a novel body plan. Integr. Comp. Biol. 46, 134–143 (2006). 21672730 10.1093/icb/icj018

[4] C. Grande, N. H. Patel, Nodal signalling is involved in left-right asymmetry in snails. Nature 457, 1007–1011 (2009). 19098895 10.1038/nature07603 PMC2661027

[5] R. J. B. Cordero, A. Casadevall, Melanin. Curr. Biol. 30, R142–R143 (2020). 32097632 10.1016/j.cub.2019.12.042

[6] S. T. Williams, Molluscan shell colour. Biol. Rev. 92, 1039–1058 (2017). 27005683 10.1111/brv.12268

[7] J. Gefaell, J. Galindo, E. Rolán-Alvarez, Shell color polymorphism in marine gastropods. Evol. Appl. 16, 202–222 (2023). 36793692 10.1111/eva.13416 PMC9923496

[8] F. Liu, B. Cai, S. Lian, X. Chang, D. Chen, Z. Pu, L. Bao, J. Wang, J. Lv, H. Zheng, Z. Bao, L. Zhang, S. Wang, Y. Li, MolluscDB 2.0: A comprehensive functional and evolutionary genomics database for over 1400 molluscan species. Nucleic Acids Res. 53, D1075–D1086 (2025). 39530242 10.1093/nar/gkae1026 PMC11701707

[9] L. N. Vandenberg, M. Levin, A unified model for left-right asymmetry? Comparison and synthesis of molecular models of embryonic laterality. Dev. Biol. 379, 1–15 (2013). 23583583 10.1016/j.ydbio.2013.03.021 PMC3698617

[10] G. Li, X. Liu, C. Xing, H. Zhang, S. M. Shimeld, Y. Wang, Cerberus-Nodal-Lefty-Pitx signaling cascade controls left-right asymmetry in amphioxus. Proc. Natl. Acad. Sci. U.S.A. 114, 3684–3689 (2017). 28320954 10.1073/pnas.1620519114 PMC5389317

[11] R. Kuroda, Left-right asymmetry in invertebrates: From molecules to organisms. Annu. Rev. Cell Dev. Biol. 40, 97–117 (2024). 38985858 10.1146/annurev-cellbio-111822-010628

[12] M. Abe, R. Kuroda, The development of CRISPR for a mollusc establishes the formin *Lsdia1* as the long-sought gene for snail dextral/sinistral coiling. Development 146, dev175976 (2019). 31088796 10.1242/dev.175976

[13] C. Bian, R. Li, Z. Wen, W. Ge, Q. Shi, Phylogenetic analysis of core melanin synthesis genes provides novel insights into the molecular basis of albinism in fish. Front. Genet. 12, 707228 (2021). 34422008 10.3389/fgene.2021.707228 PMC8371935

[14] Y. Zhu, Q. Li, H. Yu, S. Liu, L. Kong, Shell biosynthesis and pigmentation as revealed by the expression of tyrosinase and tyrosinase-like protein genes in Pacific oyster (*Crassostrea gigas*) with different shell colors. Marine Biotechnol. 23, 777–789 (2021). 10.1007/s10126-021-10063-2 34490547

[15] A. Körner, J. Pawelek, Mammalian tyrosinase catalyzes three reactions in the biosynthesis of melanin. Science 217, 1163–1165 (1982). 6810464 10.1126/science.6810464

[16] P. J. Wittkopp, S. B. Carroll, A. Kopp, Evolution in black and white: Genetic control of pigment patterns in *Drosophila*. Trends Genet. 19, 495–504 (2003). 12957543 10.1016/S0168-9525(03)00194-X

[17] S. V. Saenko, M. Schilthuizen, Evo-devo of shell colour in gastropods and bivalves. Curr. Opin. Genet. Dev. 69, 1–5 (2021). 33388521 10.1016/j.gde.2020.11.009

[18] B. Hu, H. Yu, C. Xu, L. Kong, S. Liu, Q. Li, Shell colour diversity in marine molluscs: From current knowledge to future aquaculture applications. Rev. Aquac. 17, e70038 (2025).

[19] G. Haszprunar, On the origin and evolution of major gastropod groups, with special reference to the Streptoneura. J. Moll. Stud. 54, 367–441 (1988).

[20] W. Ponder, D. R. Lindberg, *Phylogeny and Evolution of the Mollusca* (University of California Press, 2008).

[21] J. Voltzow, Gastropoda: Prosobranchia, in *Microscopic Anatomy of Invertebrates*, I. Mollusca, F. W. Harrison, A. J. Kohn, Eds. (Wiley-Liss, New York, 1994), vol. 5, pp. 111–252.

[22] S. X. Hao, Preliminary research of black mantle and muscle of the New Zealand *Haliotis iris* M.S. thesis, Shanghai Ocean University, 2018.

[23] J. Naylor, N. Andrew, S. Kim, Demographic variation in the New Zealand abalone Haliotis iris. Mar. Freshw. Res. 57, 215–224 (2006).

[24] H. J. N. Evans, H. Peters, *Haliotis iris*. The IUCN Red List Threat. Species 2021, e.T78769001A78772483 (2021).

[25] P. A. Cook, The worldwide abalone industry. Mod. Econ. 5, 1181–1186 (2014).

[26] C. I. Gallardo-Escarate, M. L. A. del Rio-Portilla, Karyotype composition in three California abalones and their relationship with genome size. J. Shellfish. Res. 26, 825–832 (2007).

[27] K. Miyaki, O. Tabeta, H. Kayano, Karyotypes of the two species of abalones *Nordotis discu*s and *N. gigantea*. Fish. Sci. 63, 179–180 (1997).

[28] G. L. Foster, C. H. Lear, J. W. B. Rae, The evolution of pCO_2_, ice volume and climate during the middle Miocene. Earth Planet. Sci. Lett. 341, 243–254 (2012).

[29] A. Holbourn, W. Kuhnt, K. G. D. Kochhann, K. M. Matsuzaki, N. Andersen, Middle Miocene climate-carbon cycle dynamics: Keys for understanding future trends on a warmer Earth? Geol. Soc. Am. Spec. Paper 556, 1–19 (2022).

[30] D. J. Hill, K. P. Bolton, A. M. Haywood, Modelled ocean changes at the Plio-Pleistocene transition driven by Antarctic ice advance. Nat. Commun. 8, 14376 (2017). 28252023 10.1038/ncomms14376 PMC5337981

[31] M. Willeit, A. Ganopolski, R. Calov, V. Brovkin, Mid-Pleistocene transition in glacial cycles explained by declining CO_2_ and regolith removal. Sci. Adv. 5, eaav7337 (2019). 30949580 10.1126/sciadv.aav7337 PMC6447376

[32] P. Gibbard, T. van Kolfschoten, The Pleistocene and Holocene epochs, in *A Geologic Time Scale 2004*, F. M. Gradstein, J. G. Ogg, A. G. Smith, Eds. (Cambridge Univ. Press, 2005), pp. 441–452.

[33] Y. Wang, X. Guo, Chromosomal rearrangement in Pectinidae revealed by rRNA loci and implications for bivalve evolution. Biol. Bull. 207, 247–256 (2004). 15616355 10.2307/1543213

[34] S. Wang, J. Zhang, W. Jiao, J. Li, X. Xun, Y. Sun, X. Guo, P. Huan, B. Dong, L. Zhang, X. Hu, X. Sun, J. Wang, C. Zhao, Y. Wang, D. Wang, X. Huang, R. Wang, J. Lv, Y. Li, Z. Zhang, B. Liu, W. Lu, Y. Hui, J. Liang, Z. Zhou, R. Hou, X. Li, Y. Liu, H. Li, X. Ning, Y. Lin, L. Zhao, Q. Xing, J. Dou, Y. Li, J. Mao, H. Guo, H. Dou, T. Li, C. Mu, W. Jiang, Q. Fu, X. Fu, Y. Miao, J. Liu, Q. Yu, R. Li, H. Liao, X. Li, Y. Kong, Z. Jiang, D. Chourrout, R. Li, Z. Bao, Scallop genome provides insights into evolution of bilaterian karyotype and development. Nat. Ecol. Evol. 1, 0120 (2017). 28812685 10.1038/s41559-017-0120 PMC10970998

[35] L. Baptista, G. Fassio, S. Gofas, M. Oliverio, S. P. Ávila, A. M. Santos, Evaluating the taxonomic status of the large sized Tricolia Risso, 1826 in the Northeast Atlantic and Mediterranean Sea. Mol. Phylogenet. Evol. 186, 107857 (2023). 37315708 10.1016/j.ympev.2023.107857

[36] V. Roussel, A. Van Wormhoudt, The effect of Pleistocene climate fluctuations on distribution of European abalone (*Haliotis tuberculata*), revealed by combined mitochondrial and nuclear marker analyses. Biochem. Genet. 55, 124–154 (2017). 27766440 10.1007/s10528-016-9778-1

[37] L. Wei, F. Xu, Y. Wang, Z. Cai, W. Yu, C. He, Q. Jiang, X. Xu, W. Guo, X. Wang, The molecular differentiation of anatomically paired left and right mantles of the Pacific oyster *Crassostrea gigas*. Mar. Biotechnol. 20, 425–435 (2018). 10.1007/s10126-018-9806-8 29594756

[38] F. Kopp, J. T. Mendell, Functional classification and experimental dissection of long noncoding RNAs. Cell 172, 393–407 (2018). 29373828 10.1016/j.cell.2018.01.011 PMC5978744

[39] J.-H. Yoon, K. Abdelmohsen, M. Gorospe, Posttranscriptional gene regulation by long noncoding RNA. J. Mol. Biol. 425, 3723–3730 (2013). 23178169 10.1016/j.jmb.2012.11.024 PMC3594629

[40] K. Yap, T. H. Chung, E. V. Makeyev, Hybridization-proximity labeling reveals spatially ordered interactions of nuclear RNA compartments. Mol. Cell 82, 463–478.e11 (2022). 34741808 10.1016/j.molcel.2021.10.009 PMC8791277

[41] V. Agarwal, G. W. Bell, J.-W. Nam, D. P. Bartel, Predicting effective microRNA target sites in mammalian mRNAs. eLife 4, e05005 (2015). 26267216 10.7554/eLife.05005 PMC4532895

[42] Q. Sun, Q. Zhou, Y. Qiao, X. Chen, H. Sun, H. Wang, Pervasive RNA-binding protein enrichment on TAD boundaries regulates TAD organization. Nucleic Acids Res. 53, gkae1271 (2025). 39777468 10.1093/nar/gkae1271 PMC11705077

[43] H. Huang, Q. Wu, Pushing the TAD boundary: Decoding insulator codes of clustered CTCF sites in 3D genomes. Bioessays 46, e2400121 (2024). 39169755 10.1002/bies.202400121

[44] S. Diederichs, The four dimensions of noncoding RNA conservation. Trends Genet. 30, 121–123 (2014). 24613441 10.1016/j.tig.2014.01.004

[45] A. Nitsche, P. F. Stadler, Evolutionary clues in lncRNAs. Wiley Interdiscip. Rev. RNA 8, e1376 (2017). 10.1002/wrna.1376 27436689

[46] G. F. Bailey, A. M. Bilsky, M. B. Rowland, A. Z. Poole, Characterization and expression of tyrosinase-like genes in the anemone *Exaiptasia pallida* as a function of health and symbiotic state. Dev. Comp. Immunol. 101, 103459 (2019). 31377102 10.1016/j.dci.2019.103459

[47] R. Esposito, S. D’Aniello, P. Squarzoni, M. R. Pezzotti, F. Ristoratore, A. Spagnuolo, New insights into the evolution of metazoan tyrosinase gene family. PLOS ONE 7, e35731 (2012). 22536431 10.1371/journal.pone.0035731 PMC3334994

[48] A. Miserez, Y. Li, J. H. Waite, F. Zok, Jumbo squid beaks: Inspiration for design of robust organic composites. Acta Biomater. 3, 139–149 (2007). 17113369 10.1016/j.actbio.2006.09.004

[49] M. Li, J. Tang, M. Yuan, B. Huang, Y. Liu, L. Wei, Y. Han, X. Zhang, X. Wang, G. Yu, X. Sang, N. Fan, S. Cai, Y. Zheng, M. Zhang, X. Wang, Outer fold is sole effective tissue among three mantle folds with regard to oyster shell colour. Int. J. Biol. Macromol. 241, 124655 (2023). 37121412 10.1016/j.ijbiomac.2023.124655

[50] Y. Tan, S. Hoon, P. A. Guerette, W. Wei, A. Ghadban, C. Hao, A. Miserez, J. H. Waite, Infiltration of chitin by protein coacervates defines the squid beak mechanical gradient. Nat. Chem. Biol. 11, 488–495 (2015). 26053298 10.1038/nchembio.1833

[51] L. Qiao, Z.-w. Yan, G. Xiong, Y.-j. Hao, R.-x. Wang, H. Hu, J.-b. Song, X.-l. Tong, L.-r. Che, S.-z. He, B. Chen, J. Mallet, C. Lu, F.-y. Dai, Excess melanin precursors rescue defective cuticular traits in stony mutant silkworms probably by upregulating four genes encoding RR1-type larval cuticular proteins. Insect Biochem. Mol. Biol. 119, 103315 (2020). 31945452 10.1016/j.ibmb.2020.103315

[52] Á. Sánchez-Ferrer, J. N. Rodríguez-López, F. García-Cánovas, F. García-Carmona, Tyrosinase: A comprehensive review of its mechanism. Biochim. Biophys. Acta 1247, 1–11 (1995). 7873577 10.1016/0167-4838(94)00204-t

[53] F. Aguilera, C. McDougall, B. M. Degnan, Evolution of the tyrosinase gene family in bivalve molluscs: Independent expansion of the mantle gene repertoire. Acta Biomater. 10, 3855–3865 (2014). 24704693 10.1016/j.actbio.2014.03.031

[54] Z. Yang, L. Zhang, J. Hu, J. Wang, Z. Bao, S. Wang, The evo-devo of molluscs: Insights from a genomic perspective. Evol. Dev. 22, 409–424 (2020). 32291964 10.1111/ede.12336

[55] C. Nielsen, Trochophora larvae: Cell-lineages, ciliary bands, and body regions. 1. Annelida and Mollusca. J. Exp. Zool. B Mol. Dev. Evol. 302, 35–68 (2004). 14760653 10.1002/jez.b.20001

[56] G. Zhang, X. Fang, X. Guo, L. Li, R. Luo, F. Xu, P. Yang, L. Zhang, X. Wang, H. Qi, Z. Xiong, H. Que, Y. Xie, P. W. H. Holland, J. Paps, Y. Zhu, F. Wu, Y. Chen, J. Wang, C. Peng, J. Meng, L. Yang, J. Liu, B. Wen, N. Zhang, Z. Huang, Q. Zhu, Y. Feng, A. Mount, D. Hedgecock, Z. Xu, Y. Liu, T. Domazet-Loso, Y. Du, X. Sun, S. Zhang, B. Liu, P. Cheng, X. Jiang, J. Li, D. Fan, W. Wang, W. Fu, T. Wang, B. Wang, J. Zhang, Z. Peng, Y. Li, N. Li, J. Wang, M. Chen, Y. He, F. Tan, X. Song, Q. Zheng, R. Huang, H. Yang, X. Du, L. Chen, M. Yang, P. M. Gaffney, S. Wang, L. Luo, Z. She, Y. Ming, W. Huang, S. Zhang, B. Huang, Y. Zhang, T. Qu, P. Ni, G. Miao, J. Wang, Q. Wang, C. E. W. Steinberg, H. Wang, N. Li, L. Qian, G. Zhang, Y. Li, H. Yang, X. Liu, J. Wang, Y. Yin, J. Wang, The oyster genome reveals stress adaptation and complexity of shell formation. Nature 490, 49–54 (2012). 22992520 10.1038/nature11413

[57] M. R. Dorwart, N. Shcheynikov, D. Yang, S. Muallem, The solute carrier 26 family of proteins in epithelial ion transport. Physiology 23, 104–114 (2008). 18400693 10.1152/physiol.00037.2007

[58] X.-D. Zhang, P. N. Thai, L. Ren, M. C. Perez Flores, H. A. Ledford, S. Park, J. H. Lee, C.-R. Sihn, C.-W. Chang, W. C. Chen, V. Timofeyev, J. Zuo, J. W. Chan, E. N. Yamoah, N. Chiamvimonvat, Prestin amplifies cardiac motor functions. Cell Rep. 35, 109097 (2021). 33951436 10.1016/j.celrep.2021.109097 PMC8720583

[59] T. J. Schaechinger, D. Oliver, Nonmammalian orthologs of prestin (SLC26A5) are electrogenic divalent/chloride anion exchangers. Proc. Natl. Acad. Sci. U.S.A. 104, 7693–7698 (2007). 17442754 10.1073/pnas.0608583104 PMC1863495

[60] S.-W. Chou, Z. Chen, S. Zhu, R. W. Davis, J. Hu, L. Liu, C. A. Fernando, K. Kindig, W. C. Brown, R. Stepanyan, B. M. McDermott Jr., A molecular basis for water motion detection by the mechanosensory lateral line of zebrafish. Nat. Commun. 8, 2234 (2017). 29269857 10.1038/s41467-017-01604-2 PMC5740173

[61] T. Weber, M. C. Göpfert, H. Winter, U. Zimmermann, H. Kohler, A. Meier, O. Hendrich, K. Rohbock, D. Robert, M. Knipper, Expression of prestin-homologous solute carrier (SLC26) in auditory organs of nonmammalian vertebrates and insects. Proc. Natl. Acad. Sci. U.S.A. 100, 7690–7695 (2003). 12782792 10.1073/pnas.1330557100 PMC164649

[62] P. Dallos, B. Fakler, Prestin, a new type of motor protein. Nat. Rev. Mol. Cell Biol. 3, 104–111 (2002). 11836512 10.1038/nrm730

[63] S. Chen, Y. Zhou, Y. Chen, J. Gu, fastp: An ultra-fast all-in-one FASTQ preprocessor. Bioinformatics 34, i884–i890 (2018). 30423086 10.1093/bioinformatics/bty560 PMC6129281

[64] G. Marçais, C. Kingsford, A fast, lock-free approach for efficient parallel counting of occurrences of k-mers. Bioinformatics 27, 764–770 (2011). 21217122 10.1093/bioinformatics/btr011 PMC3051319

[65] T. R. Ranallo-Benavidez, K. S. Jaron, M. C. Schatz, GenomeScope 2.0 and Smudgeplot for reference-free profiling of polyploid genomes. Nat. Commun. 11, 1432 (2020). 32188846 10.1038/s41467-020-14998-3 PMC7080791

[66] N. C. Durand, M. S. Shamim, I. Machol, S. S. P. Rao, M. H. Huntley, E. S. Lander, E. L. Aiden, Juicer provides a one-click system for analyzing loop-resolution Hi-C experiments. Cell Syst. 3, 95–98 (2016). 27467249 10.1016/j.cels.2016.07.002 PMC5846465

[67] O. Dudchenko, S. S. Batra, A. D. Omer, S. K. Nyquist, M. Hoeger, N. C. Durand, M. S. Shamim, I. Machol, E. S. Lander, A. P. Aiden, E. L. Aiden, De novo assembly of the *Aedes aegypti* genome using Hi-C yields chromosome-length scaffolds. Science 356, 92–95 (2017). 28336562 10.1126/science.aal3327 PMC5635820

[68] N. C. Durand, J. T. Robinson, M. S. Shamim, I. Machol, J. P. Mesirov, E. S. Lander, E. L. Aiden, Juicebox provides a visualization system for Hi-C contact maps with unlimited zoom. Cell Syst. 3, 99–101 (2016). 27467250 10.1016/j.cels.2015.07.012 PMC5596920

[69] M. Manni, M. R. Berkeley, M. Seppey, F. A. Simão, E. M. Zdobnov, BUSCO update: Novel and streamlined workflows along with broader and deeper phylogenetic coverage for scoring of eukaryotic, prokaryotic, and viral genomes. Mol. Biol. Evol. 38, 4647–4654 (2021). 34320186 10.1093/molbev/msab199 PMC8476166

[70] H. Li, R. Durbin, Fast and accurate short read alignment with Burrows-Wheeler transform. Bioinformatics 25, 1754–1760 (2009). 19451168 10.1093/bioinformatics/btp324 PMC2705234

[71] M. G. Grabherr, B. J. Haas, M. Yassour, J. Z. Levin, D. A. Thompson, I. Amit, X. Adiconis, L. Fan, R. Raychowdhury, Q. Zeng, Z. Chen, E. Mauceli, N. Hacohen, A. Gnirke, N. Rhind, F. di Palma, B. W. Birren, C. Nusbaum, K. Lindblad-Toh, N. Friedman, A. Regev, Trinity: Reconstructing a full-length transcriptome without a genome from RNA-Seq data. Nat. Biotechnol. 29, 644–652 (2011). 21572440 10.1038/nbt.1883 PMC3571712

[72] M. Tarailo-Graovac, N. Chen, Using RepeatMasker to identify repetitive elements in genomic sequences. Curr. Protoc. Bioinformatics 25, 4.10.1–4.10.14 (2009). 10.1002/0471250953.bi0410s25 19274634

[73] J. M. Flynn, R. Hubley, C. Goubert, J. Rosen, A. G. Clark, C. Feschotte, A. F. Smit, RepeatModeler2 for automated genomic discovery of transposable element families. Proc. Natl. Acad. Sci. U.S.A. 117, 9451–9457 (2020). 32300014 10.1073/pnas.1921046117 PMC7196820

[74] Z. Xu, H. Wang, LTR_FINDER: An efficient tool for the prediction of full-length LTR retrotransposons. Nucleic Acids Res. 35, W265–W268 (2007). 17485477 10.1093/nar/gkm286 PMC1933203

[75] G. Benson, Tandem repeats finder: A program to analyze DNA sequences. Nucleic Acids Res. 27, 573–580 (1999). 9862982 10.1093/nar/27.2.573 PMC148217

[76] S. Nachtweide, M. Stanke, Multi-genome annotation with AUGUSTUS. Methods Mol. Biol. 1962, 139–160 (2019). 31020558 10.1007/978-1-4939-9173-0_8

[77] C. Camacho, G. Coulouris, V. Avagyan, N. Ma, J. Papadopoulos, K. Bealer, T. L. Madden, BLAST+: Architecture and applications. BMC Bioinformatics 10, 421 (2009). 20003500 10.1186/1471-2105-10-421 PMC2803857

[78] E. Birney, M. Clamp, R. Durbin, GeneWise and genomewise. Genome Res. 14, 988–995 (2004). 15123596 10.1101/gr.1865504 PMC479130

[79] W. J. Kent, BLAT—The BLAST-like alignment tool. Genome Res. 12, 656–664 (2002). 11932250 10.1101/gr.229202 PMC187518

[80] B. J. Haas, A. L. Delcher, S. M. Mount, J. R. Wortman, R. K. Smith Jr., L. I. Hannick, R. Maiti, C. M. Ronning, D. B. Rusch, C. D. Town, S. L. Salzberg, O. White, Improving the Arabidopsis genome annotation using maximal transcript alignment assemblies. Nucleic Acids Res. 31, 5654–5666 (2003). 14500829 10.1093/nar/gkg770 PMC206470

[81] B. J. Haas, S. L. Salzberg, W. Zhu, M. Pertea, J. E. Allen, J. Orvis, O. White, C. R. Buell, J. R. Wortman, Automated eukaryotic gene structure annotation using EVidenceModeler and the Program to Assemble Spliced Alignments. Genome Biol. 9, R7 (2008). 18190707 10.1186/gb-2008-9-1-r7 PMC2395244

[82] P. Jones, D. Binns, H.-Y. Chang, M. Fraser, W. Li, C. M. Anulla, H. M. William, J. Maslen, A. Mitchell, G. Nuka, S. Pesseat, A. F. Quinn, A. Sangrador-Vegas, M. Scheremetjew, S.-Y. Yong, R. Lopez, S. Hunter, InterProScan 5: Genome-scale protein function classification. Bioinformatics 30, 1236–1240 (2014). 24451626 10.1093/bioinformatics/btu031 PMC3998142

[83] B. Buchfink, K. Reuter, H.-G. Drost, Sensitive protein alignments at tree-of-life scale using DIAMOND. Nat. Methods 18, 366–368 (2021). 33828273 10.1038/s41592-021-01101-x PMC8026399

[84] L.-T. Nguyen, H. A. Schmidt, A. Von Haeseler, B. Q. Minh, IQ-TREE: A fast and effective stochastic algorithm for estimating maximum-likelihood phylogenies. Mol. Biol. Evol. 32, 268–274 (2015). 25371430 10.1093/molbev/msu300 PMC4271533

[85] R. C. Edgar, MUSCLE: Multiple sequence alignment with high accuracy and high throughput. Nucleic Acids Res. 32, 1792–1797 (2004). 15034147 10.1093/nar/gkh340 PMC390337

[86] M. Suyama, D. Torrents, P. Bork, PAL2NAL: Robust conversion of protein sequence alignments into the corresponding codon alignments. Nucleic Acids Res. 34, W609–W612 (2006). 16845082 10.1093/nar/gkl315 PMC1538804

[87] S. Mirarab, R. Reaz, M. S. Bayzid, T. Zimmermann, M. S. Swenson, T. Warnow, ASTRAL: Genome-scale coalescent-based species tree estimation. Bioinformatics 30, i541–i548 (2014). 25161245 10.1093/bioinformatics/btu462 PMC4147915

[88] Z. Yang, PAML 4: Phylogenetic analysis by maximum likelihood. Mol. Biol. Evol. 24, 1586–1591 (2007). 17483113 10.1093/molbev/msm088

[89] S. Kumar, M. Suleski, J. M. Craig, A. E. Kasprowicz, M. Sanderford, M. Li, G. Stecher, S. B. Hedges, TimeTree 5: An expanded resource for species divergence times. Mol. Biol. Evol. 39, msac174 (2022). 35932227 10.1093/molbev/msac174 PMC9400175

[90] Y. Lan, J. Sun, C. Chen, Y. Sun, Y. Zhou, Y. Yang, W. Zhang, R. Li, K. Zhou, W. C. Wong, Y. H. Kwan, A. Cheng, S. Bougouffa, C. L. Van Dover, J.-W. Qiu, P.-Y. Qian, Hologenome analysis reveals dual symbiosis in the deep-sea hydrothermal vent snail *Gigantopelta aegis*. Nat. Commun. 12, 1165 (2021). 33608555 10.1038/s41467-021-21450-7 PMC7895826

[91] M. J. Sanderson, r8s: Inferring absolute rates of molecular evolution and divergence times in the absence of a molecular clock. Bioinformatics 19, 301–302 (2003). 12538260 10.1093/bioinformatics/19.2.301

[92] H. Li, B. Handsaker, A. Wysoker, T. Fennell, J. Ruan, N. Homer, G. Marth, G. Abecasis, R. Durbin, 1000 Genome Project Data Processing Subgroup, The sequence alignment/map format and SAMtools. Bioinformatics 25, 2078–2079 (2009). 19505943 10.1093/bioinformatics/btp352 PMC2723002

[93] H. Li, R. Durbin, Inference of human population history from individual whole-genome sequences. Nature 475, 493–496 (2011). 21753753 10.1038/nature10231 PMC3154645

[94] G. C. Poore, Ecology of New Zealand abalones, *Haliotis* species (Mollusca: Gastropoda) 4. Reproduction. N. Z. J. Mar. Freshw. Res. 7, 67–84 (1973).

[95] Y. Wang, H. Tang, J. D. DeBarry, X. Tan, J. Li, X. Wang, T.-H. Lee, H. Jin, B. Marler, H. Guo, J. C. Kissinger, A. H. Paterson, MCScanX: A toolkit for detection and evolutionary analysis of gene synteny and collinearity. Nucleic Acids Res. 40, e49 (2012). 22217600 10.1093/nar/gkr1293 PMC3326336

[96] B. R. Jones, A. Rajaraman, E. Tannier, C. Chauve, ANGES: Reconstructing ANcestral GEnomeS maps. Bioinformatics 28, 2388–2390 (2012). 22820205 10.1093/bioinformatics/bts457

[97] D. M. Emms, S. Kelly, OrthoFinder: Solving fundamental biases in whole genome comparisons dramatically improves orthogroup inference accuracy. Genome Biol. 16, 157 (2015). 26243257 10.1186/s13059-015-0721-2 PMC4531804

[98] T. De Bie, N. Cristianini, J. P. Demuth, M. W. Hahn, CAFE: A computational tool for the study of gene family evolution. Bioinformatics 22, 1269–1271 (2006). 16543274 10.1093/bioinformatics/btl097

[99] M. Li, B. Wu, P. Zhang, Y. Li, W. Xu, K. Wang, Q. Qiu, J. Zhang, J. Li, C. Zhang, J. Fan, C. Feng, Z. Chen, Genomes of two flying squid species provide novel insights into adaptations of cephalopods to pelagic life. Genomics Proteomics Bioinformatics 20, 1053–1065 (2022). 36216027 10.1016/j.gpb.2022.09.009 PMC10225486

[100] N. Servant, N. Varoquaux, B. R. Lajoie, E. Viara, C.-J. Chen, J.-P. Vert, E. Heard, J. Dekker, E. Barillot, HiC-Pro: An optimized and flexible pipeline for Hi-C data processing. Genome Biol. 16, 259 (2015). 26619908 10.1186/s13059-015-0831-x PMC4665391

[101] J. Wolff, L. Rabbani, R. Gilsbach, G. Richard, T. Manke, R. Backofen, B. A. Grüning, Galaxy HiCExplorer 3: A web server for reproducible Hi-C, capture Hi-C and single-cell Hi-C data analysis, quality control and visualization. Nucleic Acids Res. 48, W177–W184 (2020). 32301980 10.1093/nar/gkaa220 PMC7319437

[102] L. Lopez-Delisle, L. Rabbani, J. Wolff, V. Bhardwaj, R. Backofen, B. Grüning, F. Ramírez, T. Manke, pyGenomeTracks: Reproducible plots for multivariate genomic datasets. Bioinformatics 37, 422–423 (2021). 32745185 10.1093/bioinformatics/btaa692 PMC8058774

[103] D. Kim, J. M. Paggi, C. Park, C. Bennett, S. L. Salzberg, Graph-based genome alignment and genotyping with HISAT2 and HISAT-genotype. Nat. Biotechnol. 37, 907–915 (2019). 31375807 10.1038/s41587-019-0201-4 PMC7605509

[104] S. Kovaka, A. V. Zimin, G. M. Pertea, R. Razaghi, S. L. Salzberg, M. Pertea, Transcriptome assembly from long-read RNA-seq alignments with StringTie2. Genome Biol. 20, 278 (2019). 31842956 10.1186/s13059-019-1910-1 PMC6912988

[105] M. I. Love, W. Huber, S. Anders, Moderated estimation of fold change and dispersion for RNA-seq data with DESeq2. Genome Biol. 15, 550 (2014). 25516281 10.1186/s13059-014-0550-8 PMC4302049

[106] F. Krueger, S. R. Andrews, Bismark: A flexible aligner and methylation caller for Bisulfite-Seq applications. Bioinformatics 27, 1571–1572 (2011). 21493656 10.1093/bioinformatics/btr167 PMC3102221

[107] B. Langmead, S. L. Salzberg, Fast gapped-read alignment with Bowtie 2. Nat. Methods 9, 357–359 (2012). 22388286 10.1038/nmeth.1923 PMC3322381

[108] A. Akalin, M. Kormaksson, S. Li, F. E. Garrett-Bakelman, M. E. Figueroa, A. Melnick, C. E. Mason, methylKit: A comprehensive R package for the analysis of genome-wide DNA methylation profiles. Genome Biol. 13, R87 (2012). 23034086 10.1186/gb-2012-13-10-r87 PMC3491415

[109] Y. Li, M. Hu, Z. Zhang, B. Wu, J. Zheng, F. Zhang, J. Hao, T. Xue, Z. Li, C. Zhu, Y. Liu, L. Zhao, W. Xu, P. Xin, C. Feng, W. Wang, Y. Zhao, Q. Qiu, K. Wang, Origin and stepwise evolution of vertebrate lungs. Nat. Ecol. Evol. 9, 672–691 (2025). 39953253 10.1038/s41559-025-02642-6

[110] J. Y. Kishi, S. W. Lapan, B. J. Beliveau, E. R. West, A. Zhu, H. M. Sasaki, S. K. Saka, Y. Wang, C. L. Cepko, P. Yin, SABER amplifies FISH: Enhanced multiplexed imaging of RNA and DNA in cells and tissues. Nat. Methods 16, 533–544 (2019). 31110282 10.1038/s41592-019-0404-0 PMC6544483

[111] K. S. Barratt, R. M. Arkell, Production of digoxigenin-labeled riboprobes for in situ hybridization experiments. Curr. Protoc. Mouse Biol. 10, e74 (2020). 32436648 10.1002/cpmo.74

[112] P. Huan, Q. Wang, S. Tan, B. Liu, Dorsoventral decoupling of Hox gene expression underpins the diversification of molluscs. Proc. Natl. Acad. Sci. U.S.A. 117, 503–512 (2020). 31871200 10.1073/pnas.1907328117 PMC6955316

[113] M. Martin, Cutadapt removes adapter sequences from high-throughput sequencing reads. EMBnet.J 17, 10–12 (2011).

[114] A. R. Quinlan, I. M. Hall, BEDTools: A flexible suite of utilities for comparing genomic features. Bioinformatics 26, 841–842 (2010). 20110278 10.1093/bioinformatics/btq033 PMC2832824

