# Supplementary material for: Abalone genomics reveals an ancient asymmetry axis and the multifunctionality of the mantle
Source: Sci Adv. 2026 Sep 4;12(36):eaeb8392. doi: 10.1126/sciadv.aeb8392 (PMC13544226; doi:10.1126/sciadv.aeb8392)
Supplement: Supplementary file 1 [file sciadv.aeb8392_sm.pdf]

Science Advances
AAAS

Supplementary Materials for

Abalone genomics reveals an ancient asymmetry axis and the multifunctionality of the mantle

Xiaotong Wang *et al.*

Corresponding author: Xiaotong Wang, wangxiaotong999@163.com; Kun Wang, wangkun@nwpu.edu.cn; Ximing Guo, xguo@rutgers.edu; Chenguang Feng, fengcg@nwpu.edu.cn

Sci. Adv. **12**, eaeb8392 (2026)
DOI: 10.1126/sciadv.aeb8392

The PDF file includes:

Figs. S1 to S15
Legends for tables S1 to S30

Other Supplementary Material for this manuscript includes the following:

Tables S1 to S30

Supplementary Figures

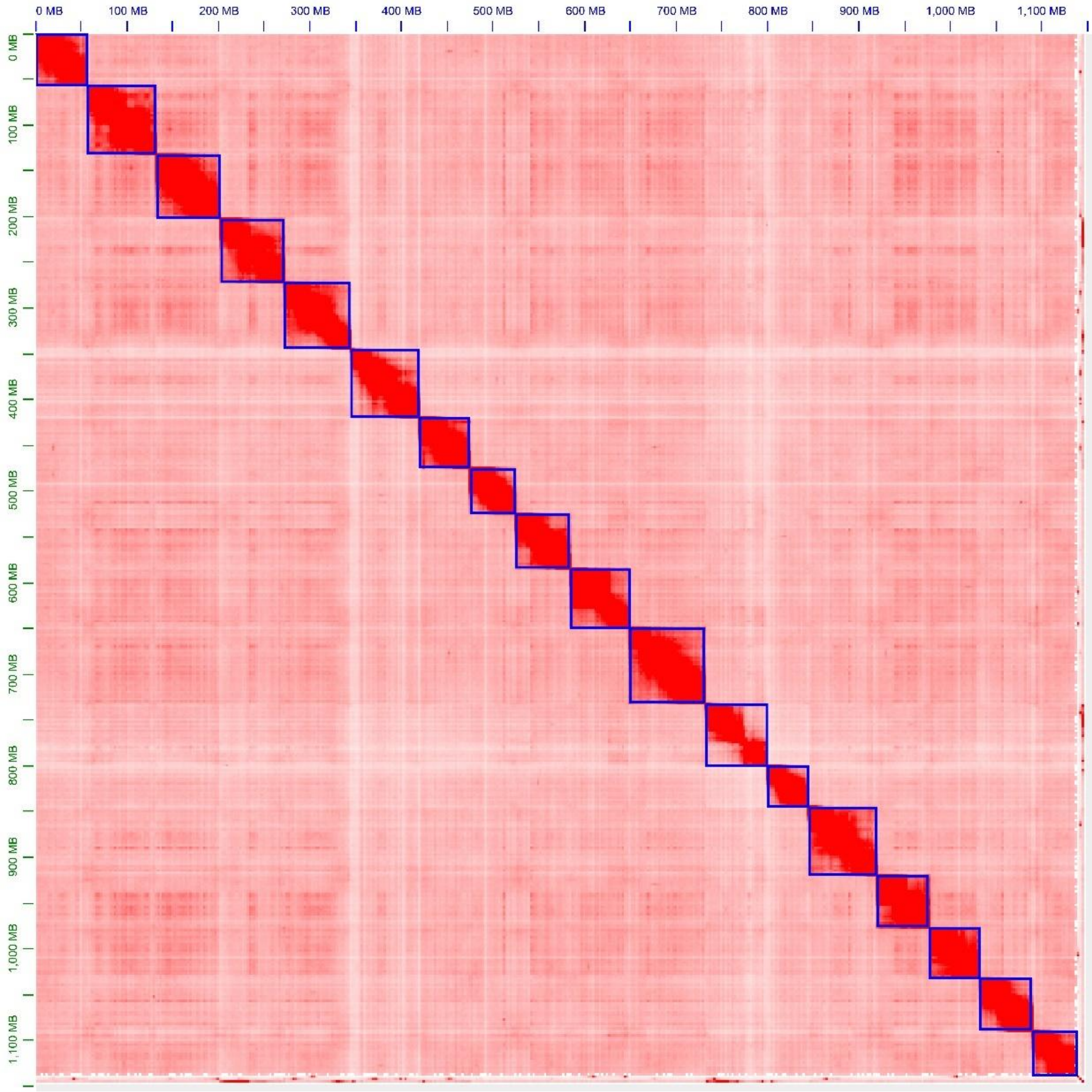

fig. S1. Hi-C linkage density map of the 18 *pseudo*-chromosomes in the assembled *Haliotis iris* genome.

The heatmap shows genome-wide Hi-C contact frequencies among the assembled scaffolds. Blue boxes delimit the 18 anchored pseudochromosomes, and the axes indicate cumulative genomic coordinates in megabases. Color intensity represents the log-scaled number of valid Hi-C read pairs within 500-kb genomic windows.

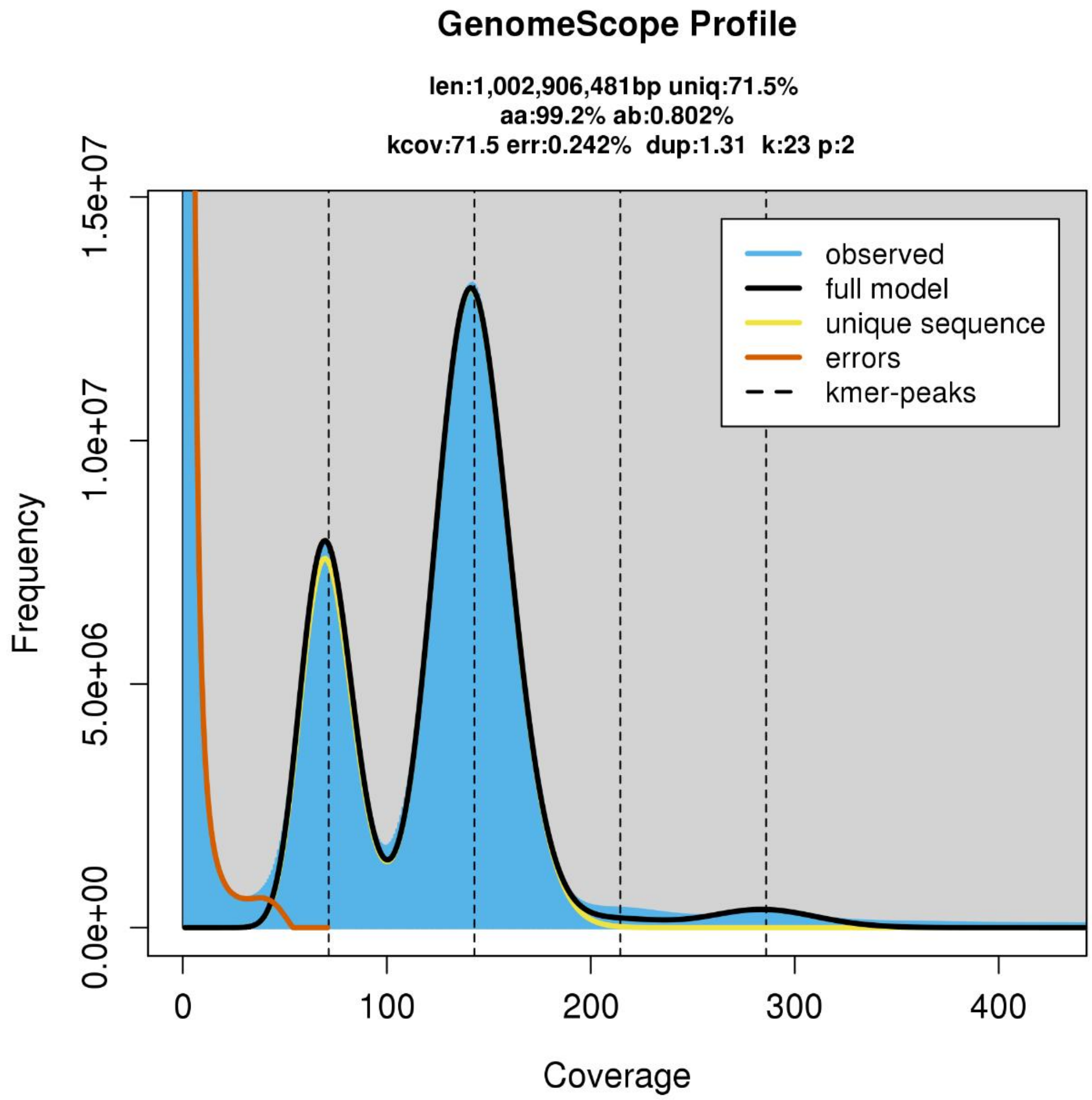

fig. S2. GenomeScope profile of the rainbow abalone genome based on 23-mer frequency analysis.

The 23-mer frequency distribution was generated from Illumina short reads using Jellyfish and modeled with GenomeScope v2.0. The x-axis indicates k-mer coverage, and the y-axis indicates k-mer frequency. The blue area shows the observed k-mer distribution, the black line shows the fitted GenomeScope model, the yellow line indicates the unique-sequence component, the orange line indicates sequencing errors, and dashed vertical lines mark inferred k-mer coverage peaks. GenomeScope estimated a genome size of 1,002,906,481 bp and a heterozygosity rate of 0.802% for *Haliotis iris*.

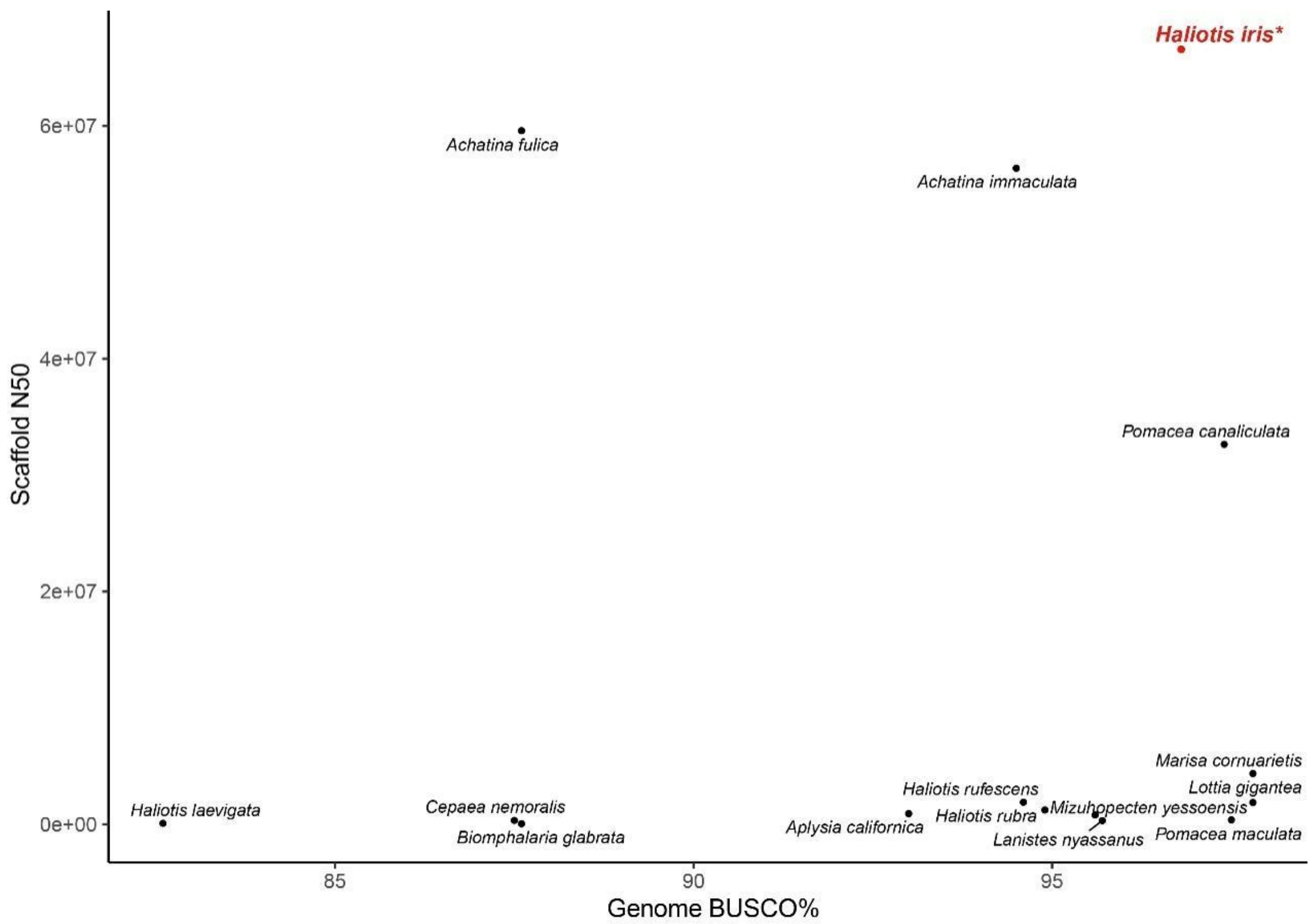

fig. S3. Genome assembly quality comparison between the rainbow abalone and other molluscan genomes.

Each point represents one molluscan genome assembly. The x-axis shows genome completeness assessed by BUSCO using the ‘metazoa_odb10’ dataset, and the y-axis shows scaffold N50 length. The rainbow abalone genome assembled in this study is highlighted in red with an asterisk. Compared with other representative molluscan genomes, the *Haliotis iris* assembly shows both high BUSCO completeness and a large scaffold N50, supporting its chromosome-level assembly quality.

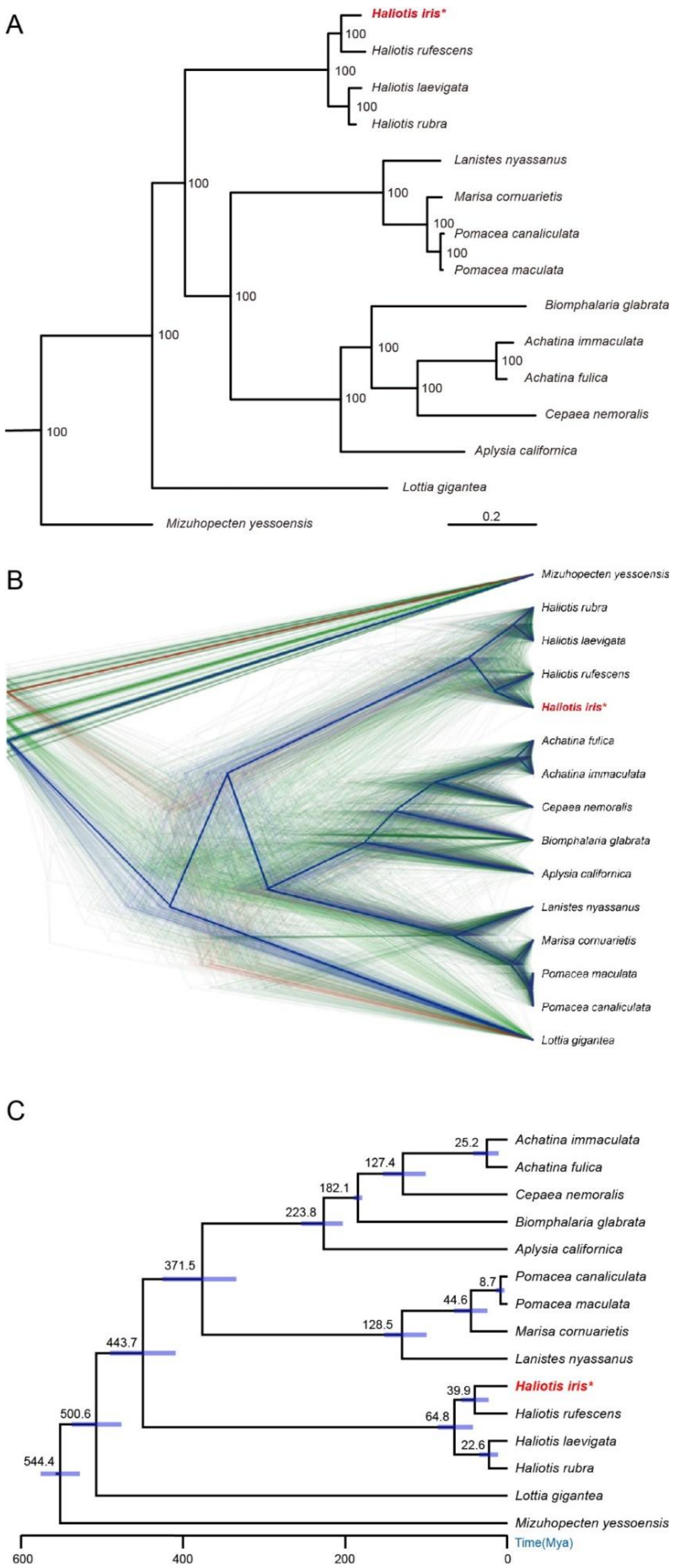

fig. S4. Phylogenetic relationships and divergence-time estimation of selected mollusks.

(A) Maximum-likelihood phylogenetic tree reconstructed from 725 single-copy

orthologous genes, with *Mizuhopecten yessoensis* used as the outgroup. Numbers at nodes indicate branch support values, and the scale bar represents substitutions per site. *Haliotis iris* is highlighted in red with an asterisk. (B) DensiTree visualization of gene-tree or species-tree topology concordance across the analyzed orthologous gene set. Darker regions indicate greater consistency in topology and branch length among trees, whereas lighter regions indicate lower concordance. (C) Time-calibrated phylogenetic tree estimated using MCMCtree. Numbers at nodes indicate estimated divergence times in million years ago (Mya), and blue horizontal bars indicate the 95% confidence intervals of estimated node ages. *H. iris* is highlighted in red with an asterisk.

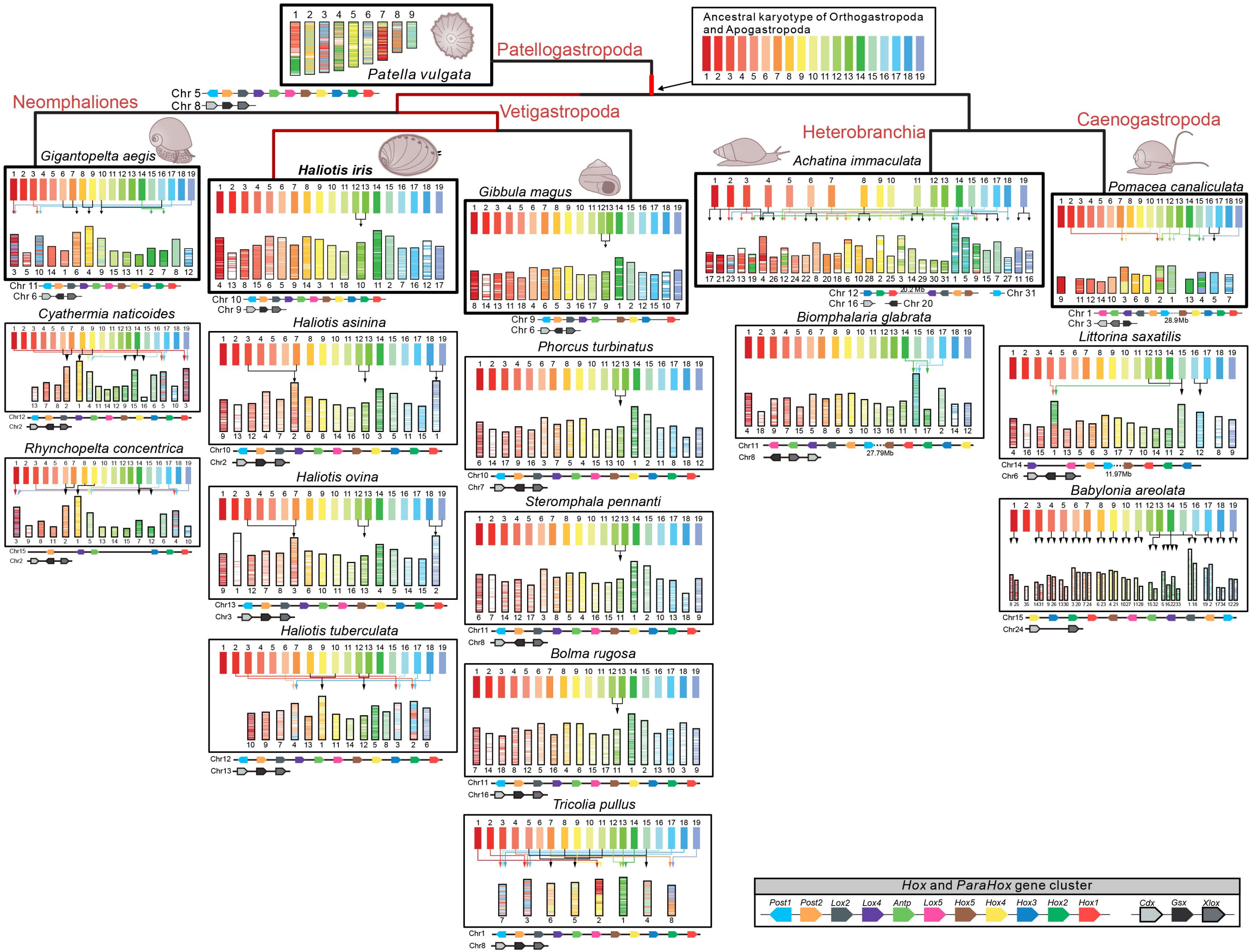

fig. S5. Expanded comparison of ancestral proto-chromosomal fragments and *Hox/ParaHox* gene organization across 18 representative gastropod genomes.

Species are arranged according to the phylogenetic topology shown in Fig. 1C, with major gastropod clades labeled along the tree. The ancestral karyotype of Orthogastropoda and Apogastropoda is shown at the top, with 19 inferred ancestral proto-chromosomal fragments numbered and color-coded. For each species, colored blocks indicate the distribution of these ancestral proto-chromosomal fragments across extant chromosomes, illustrating lineage-specific chromosomal conservation, fusion, fission, and rearrangement patterns. Gene tracks below each species panel show the chromosomal organization of *Hox* and *ParaHox* genes, with gene identities indicated by the color legend at the bottom. Bold branches and boxed panels indicate the six representative species summarized in Fig. 1C.

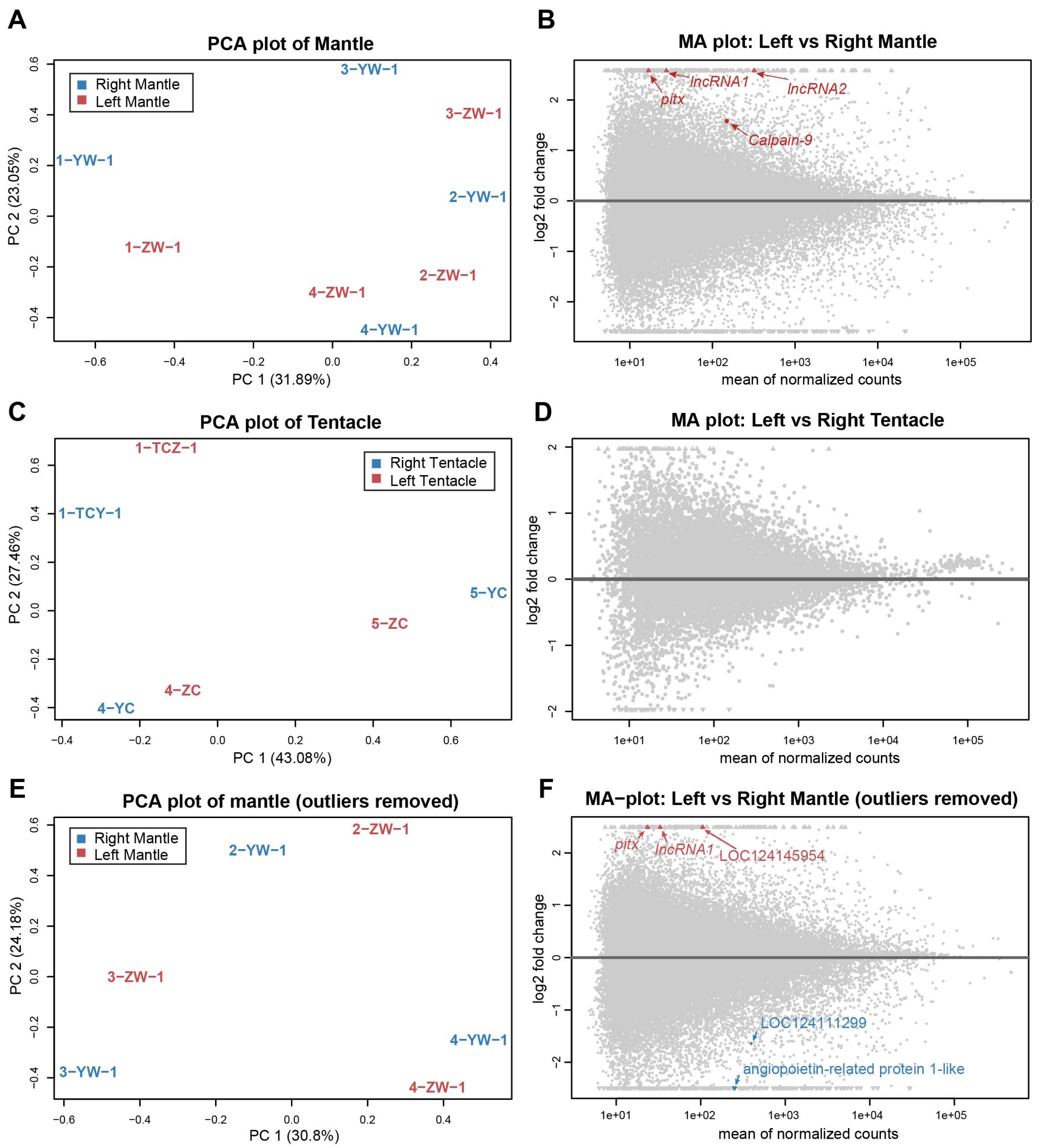

fig. S6. PCA and MA plots of paired mantle and cephalic tentacle transcriptomes. (A) PCA plot of left and right mantle lobe transcriptomes using all four individuals. (B) MA plot for the left versus right mantle lobe comparison using all four individuals. (C) PCA plot of left and right cephalic tentacle transcriptomes. (D) MA plot for the left versus right cephalic tentacle comparison. (E) PCA plot of the mantle lobe transcriptomes after excluding individual 1, whose paired mantle samples (1-ZW-1 and 1-YW-1) showed relatively separated clustering in panel A. (F) MA plot for the left versus right mantle lobe comparison after excluding individual 1. Significantly differentially expressed candidates identified in the MA analyses are highlighted and labeled in red or blue. The paired cephalic tentacles showed no statistically significant

left-right transcriptional divergence, whereas the paired mantle lobes showed only a limited asymmetry-associated signal. In the original mantle analysis, *pitx*, *lncRNA1*, *lncRNA2*, and *Calpain-9* were among the most prominent candidates, with *Calpain-9* detected near the significance threshold. After excluding individual 1, *pitx* and *lncRNA1* remained prominent left-biased candidates, whereas several additional candidates were detected in the reanalysis. Sample numbers and tissue information corresponding to the labels in the PCA plots are provided in table S1.

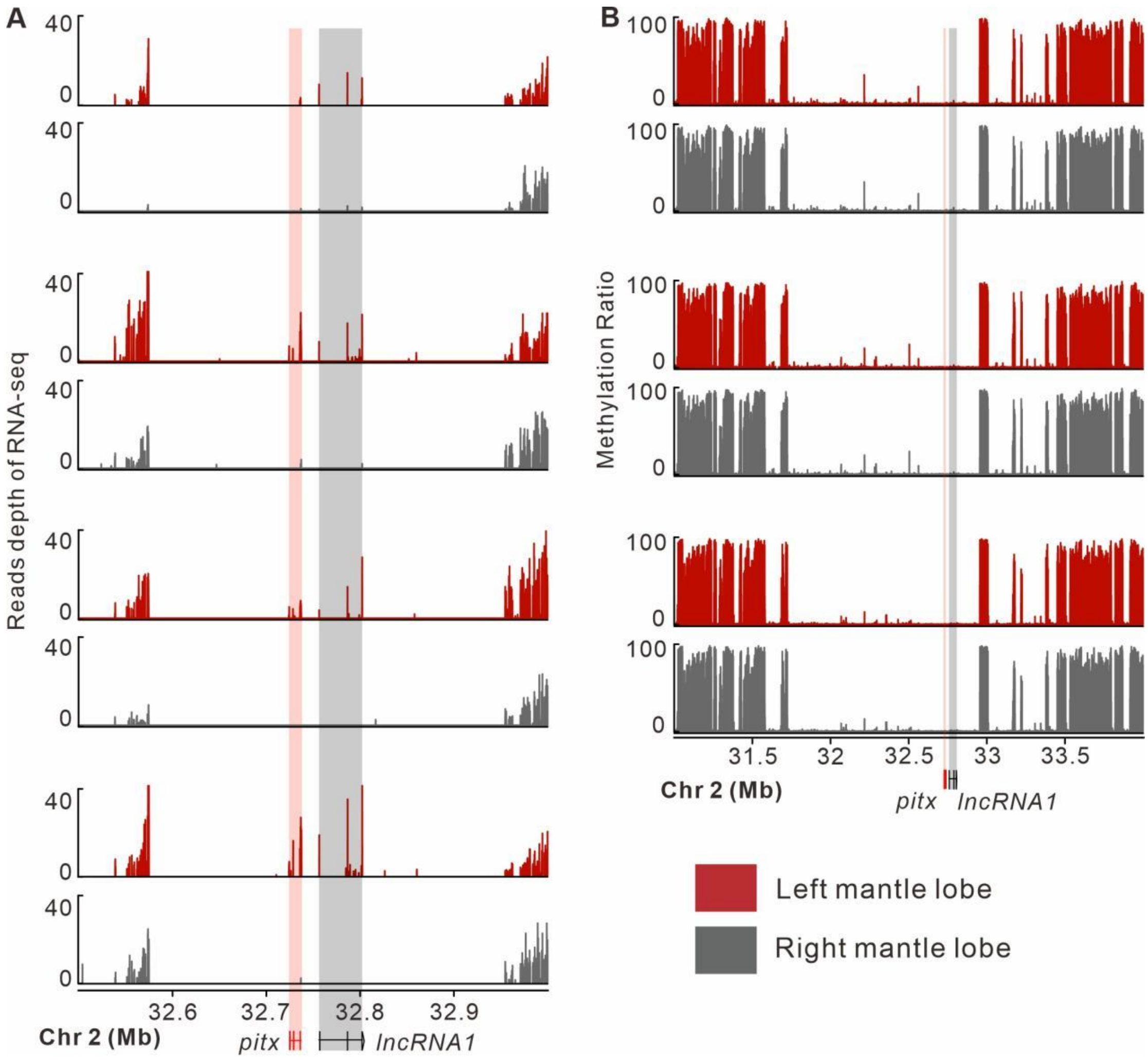

fig. S7. Replicate RNA-seq and DNA methylation tracks around the *pitx-lncRNA1* locus.

(A) RNA-seq read-depth tracks across the Chr2 region surrounding *pitx* and *lncRNA1* in paired left and right mantle lobes. Individual biological replicates are shown separately (n = 4). (B) DNA methylation ratio tracks across the same genomic region in paired left and right mantle lobes. Individual biological replicates are shown separately (n = 3). Left-mantle tracks are shown in red, and right-mantle tracks are shown in gray. Vertical shaded regions mark the positions of *pitx* and *lncRNA1*. These replicate-level tracks correspond to the summarized profiles shown in Fig. 2C and illustrate left-biased expression of the *pitx-lncRNA1* region without an obvious corresponding left-right difference in local DNA methylation.

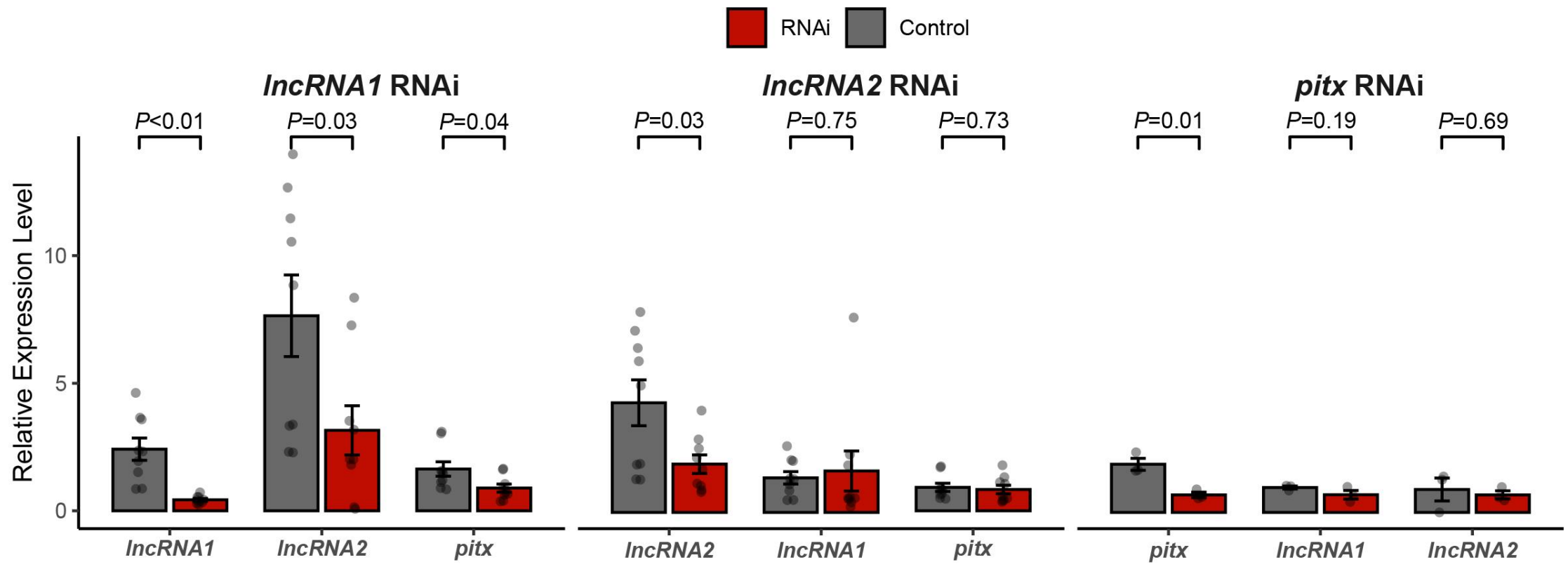

fig. S8. Reciprocal RNAi analysis of the *lncRNA1–lncRNA2–pitx* regulatory module in *H. iris*.

qRT-PCR analysis of *lncRNA1*, *lncRNA2*, and *pitx* expression after RNAi-mediated knockdown of each gene in mantle tissue. Relative expression was calculated using the $2^{-\Delta\Delta Ct}$ method and normalized to the corresponding control group. Data are shown as mean ± SEM; dots represent individual biological replicates. For *lncRNA1* and *lncRNA2* RNAi, $n = 9$ biological replicates per group; for *pitx* RNAi, $n = 3$ biological replicates per group. Statistical significance was assessed using independent-samples *t* tests, with *P* values indicated above the corresponding comparisons. Knockdown of *lncRNA1* significantly reduced both *lncRNA2* and *pitx* expression, whereas knockdown of *lncRNA2* or *pitx* did not significantly alter the other two genes. These results support a directional regulatory relationship from *lncRNA1* toward *pitx* expression.

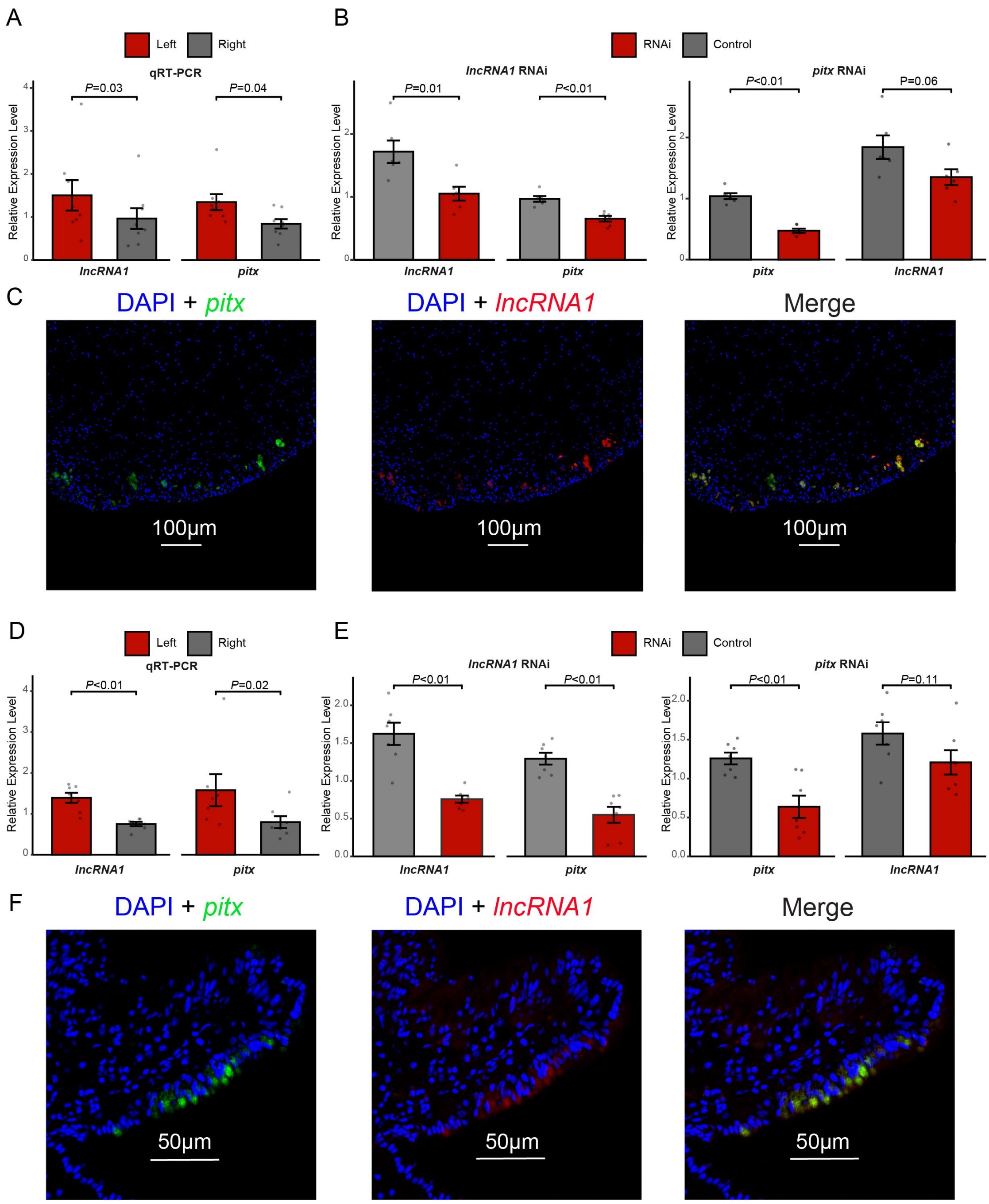

fig. S9. Cross-species functional validation of the conserved *pitx-lncRNA1* regulatory association in the limpet *Cellana toreuma* and the trochid *Monodonta labio*.

(A-C) Functional and spatial characterization in *C. toreuma*. (A) qRT-PCR showing higher expression of *pitx* and *lncRNA1* in the left mantle than in the right mantle, consistent with the pattern observed in *H. iris*. Relative expression was calculated

using the $2^{-\Delta\Delta Ct}$ method. Data are shown as mean ± SEM; $n = 8$ biological replicates. Statistical significance was assessed using paired *t* tests. (B) RNAi assays showing that knockdown of *lncRNA1* significantly reduces *pitx* expression, whereas knockdown of *pitx* does not significantly alter *lncRNA1* expression. Data are shown as mean ± SEM; $n = 6$ biological replicates per group. Statistical significance was assessed using independent-samples *t* tests. (C) *In situ* hybridization showing strong co-localization of *pitx* and *lncRNA1* in mantle tissue. (D-F) Functional and spatial characterization in *Monodonta labio*. (D) qRT-PCR showing higher expression of *pitx* and *lncRNA1* in the left mantle than in the right mantle, consistent with the pattern observed in *H. iris*. Relative expression was calculated using the $2^{-\Delta\Delta Ct}$ method. Data are shown as mean ± SEM; $n = 7$ biological replicates. Statistical significance was assessed using paired *t* tests. (E) RNAi assays showing that knockdown of *lncRNA1* significantly reduces *pitx* expression, whereas knockdown of *pitx* does not significantly alter *lncRNA1* expression. Data are shown as mean ± SEM; $n = 7$ biological replicates per group. Statistical significance was assessed using independent-samples *t* tests. (F) *In situ* hybridization showing strong co-localization of *pitx* and *lncRNA1* in mantle tissue. Together, these results provide functional support for the broader conservation of the *pitx-lncRNA1* regulatory association suggested by the comparative synteny analysis in Fig. 4C.

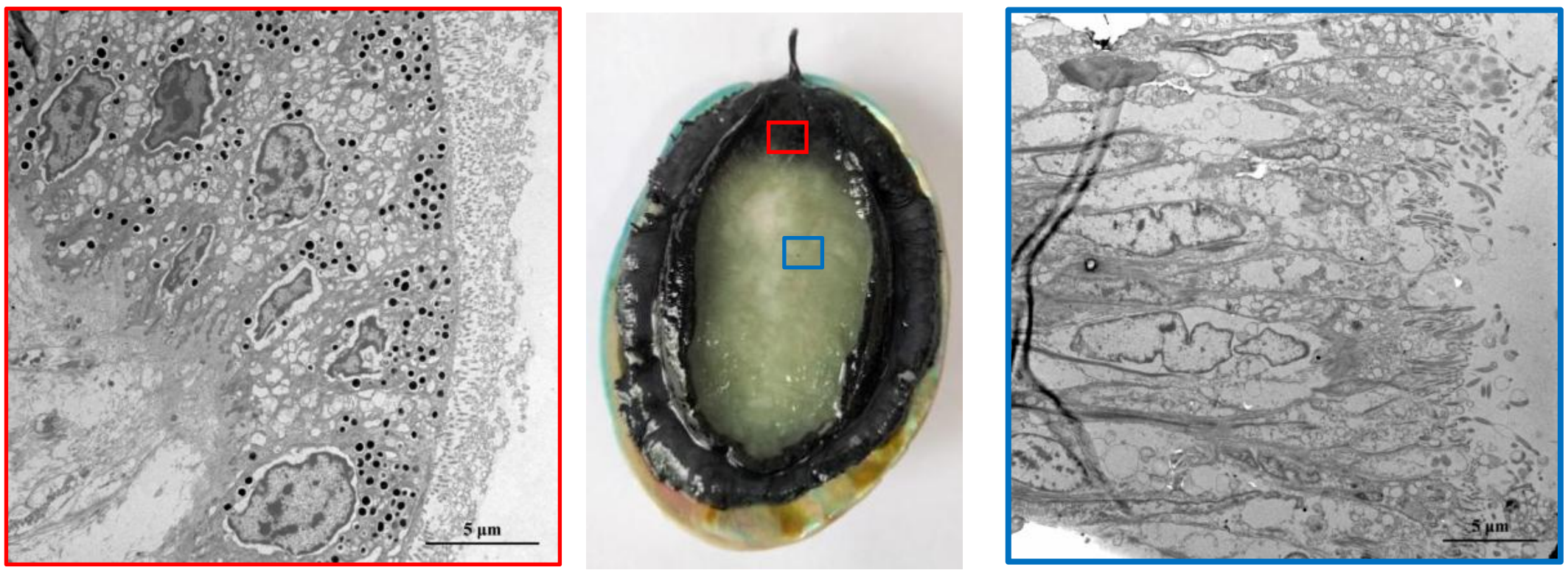

fig. S10. Transmission electron micrographs comparing melanin distribution in pigmented and unpigmented pedal regions of the rainbow abalone.

The central photograph, reproduced from Fig. 5A, shows the macroscopic contrast between the pigmented lower pedal epithelium and the unpigmented lower pedal region. Red and blue boxes indicate the sampled regions corresponding to the left and right TEM images, respectively. The left TEM image shows abundant electron-dense melanin particles in the pigmented epithelium, whereas the right TEM image shows no obvious melanin particles in the unpigmented region. The left TEM image is the same as the fourth panel in Fig. 5J and is reproduced here for direct comparison with the unpigmented region. Scale bars, 5 µm.

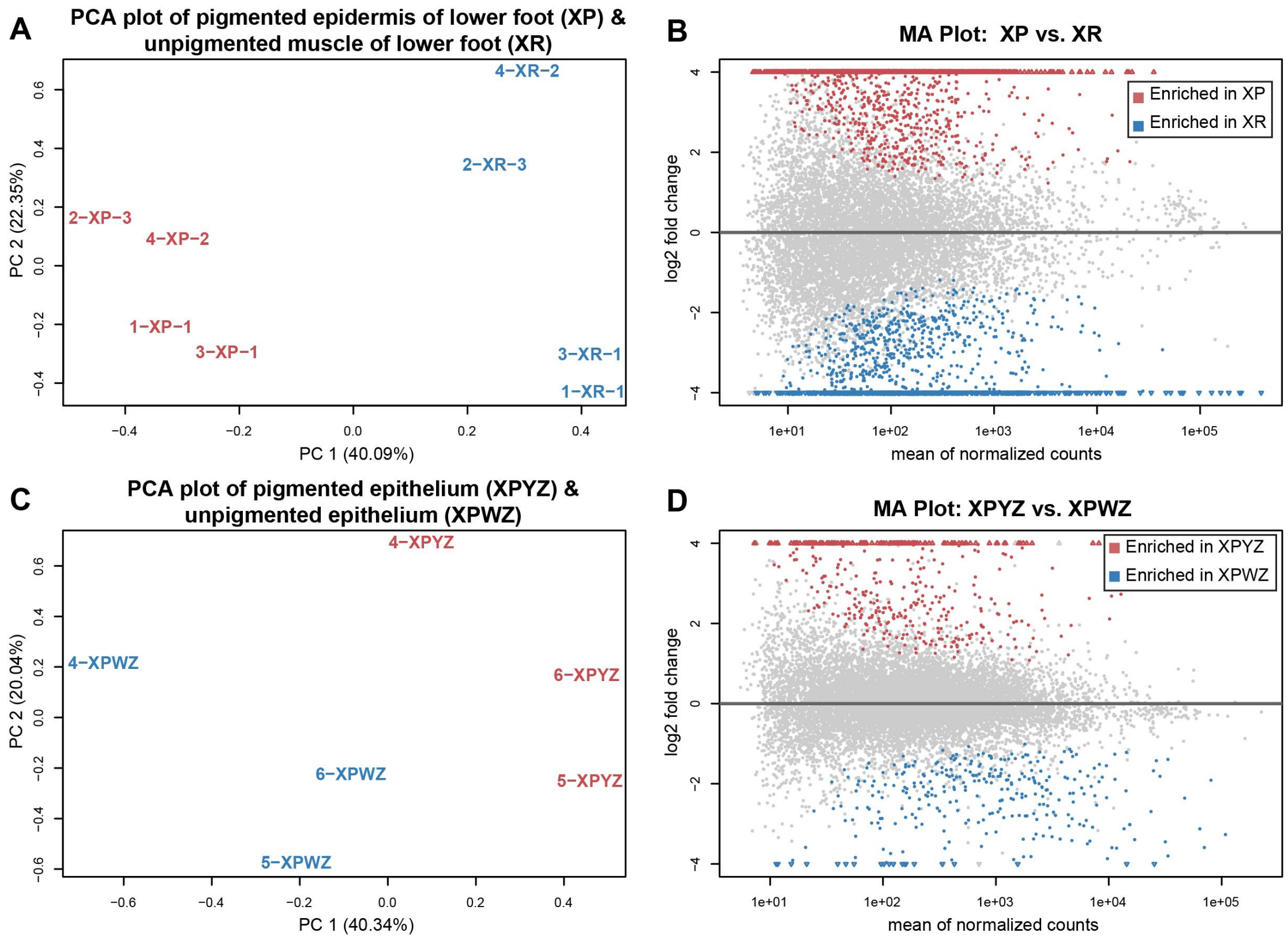

fig. S11. PCA and MA plots of melanin-associated sample transcriptomes.

(A) PCA plot of pigmented epidermis of the lower foot (XP) and unpigmented muscle of the lower foot (XR). (B) MA plot for the XP versus XR comparison. (C) PCA plot of pigmented epithelium (XPYZ) and unpigmented epithelium (XPWZ). (D) MA plot for the XPYZ versus XPWZ comparison. In the MA plots, differentially expressed genes (DEGs) are highlighted in color: red indicates genes enriched in the pigmented samples (XP or XPYZ), and blue indicates genes enriched in the corresponding unpigmented samples (XR or XPWZ). DEGs were defined using the adjusted *P*-value threshold described in the Methods. Triangles indicate data points outside the displayed y-axis range. Tissue information corresponding to the numerical sample labels in panels A and C is provided in table S1.

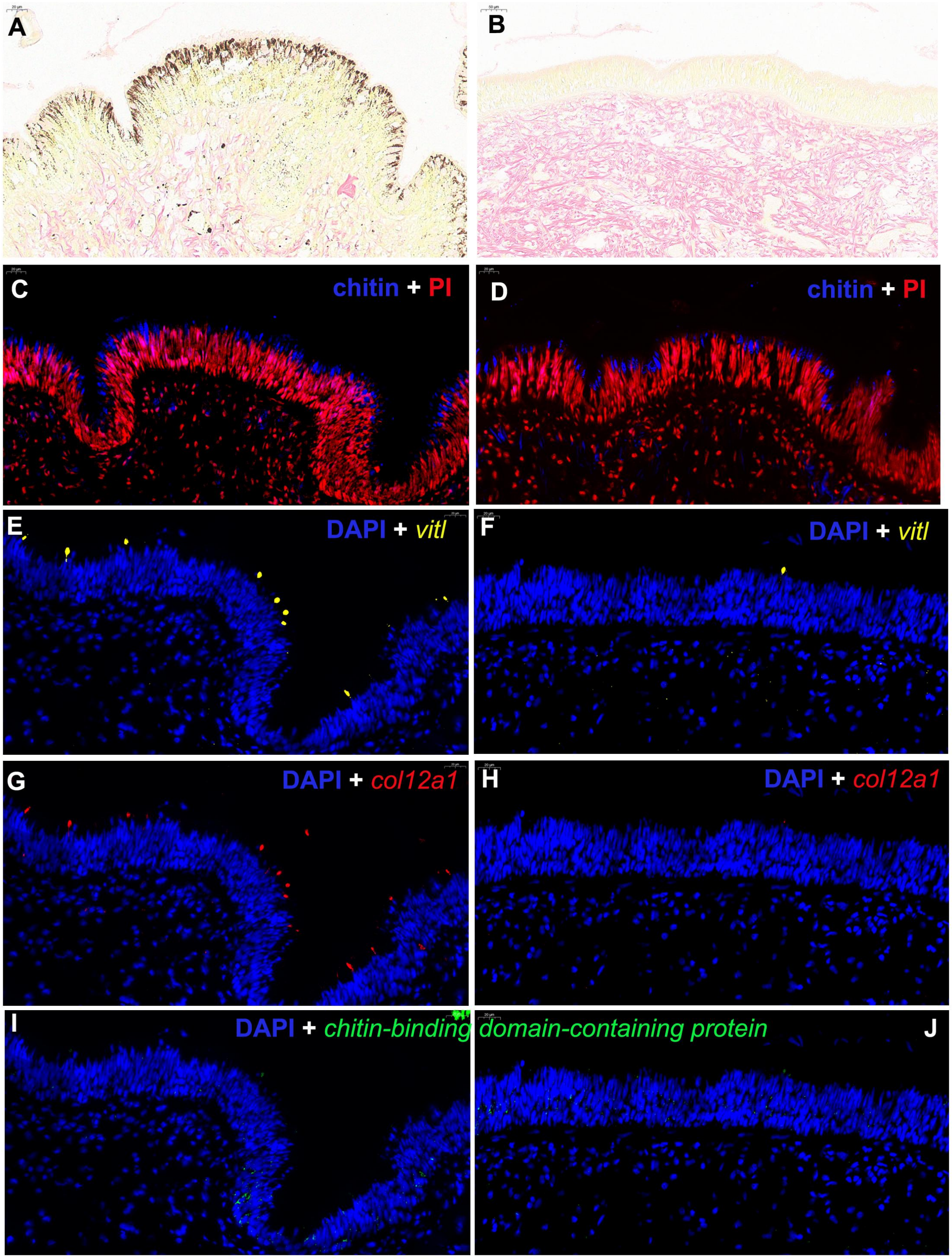

fig. S12. Histological and spatial association of melanin deposition with chitin signal and chitin-binding-related gene expression in Haliotis iris pedal epithelium. (A and B) Melanin staining of pigmented epithelium from color-variant individuals (PE2; A) and unpigmented epithelium from the same region (UE; B). (C and D) Chitin staining in PE2 (C) and UE (D). Chitin signal is shown in blue, and PI marks nuclei in red. (E and F) Fluorescent in situ hybridization of *vitl* in PE2 (E) and UE (F).

(G and H) Fluorescent in situ hybridization of *col12a1* in PE2 (G) and UE (H). (I and J) Fluorescent in situ hybridization of a *chitin-binding domain-containing protein* in PE2 (I) and UE (J). For in situ hybridization panels, DAPI marks nuclei in blue, and gene-specific signals are shown in the colors indicated in each panel. These representative sections show that melanin-rich epithelium is accompanied by stronger chitin signal and localized expression of selected chitin-associated genes. These observations support, but do not directly prove, the hypothesis that chitin-associated matrices contribute to melanin deposition in *H. iris*.

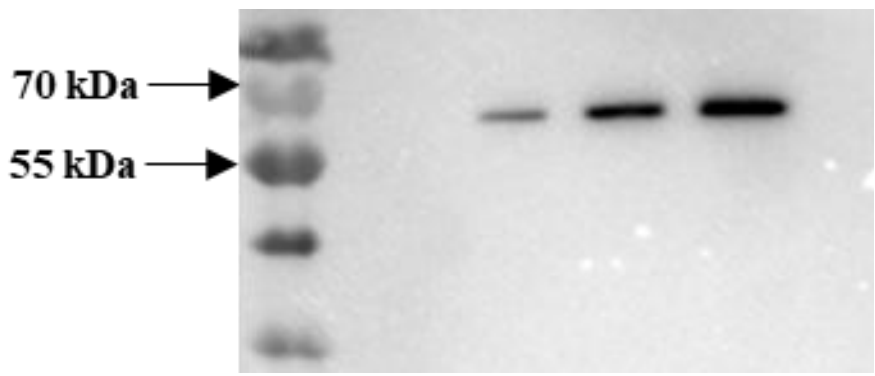

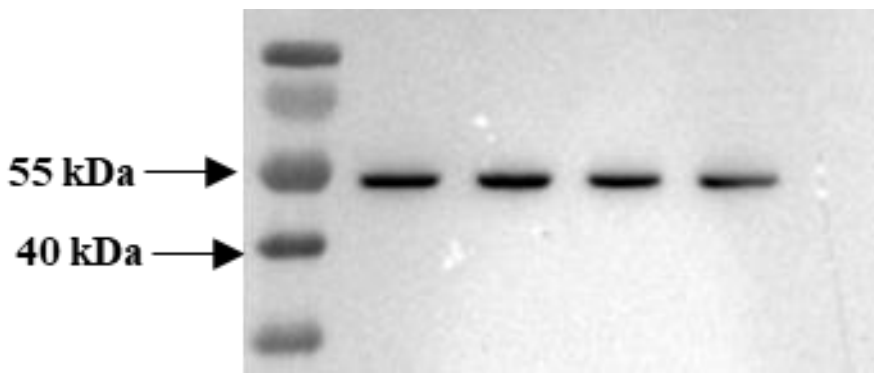

fig. S13. Immunoblot source data for the dual-luciferase reporter assay shown in Fig. 5F.

Representative Western blots showing MITF-Myc protein expression (left) and *β*-tubulin loading control (right) in HEK293T cells used for the reporter assay. Lanes correspond to increasing amounts of transfected *mitf*-Myc plasmid (0, 100, 200, and 400 ng), as indicated in Fig. 5F. The MITF-Myc signal increased with increasing plasmid dose, whereas *β*-tubulin remained relatively stable across samples, supporting comparable protein loading. Molecular weight markers are indicated on the left of each blot.

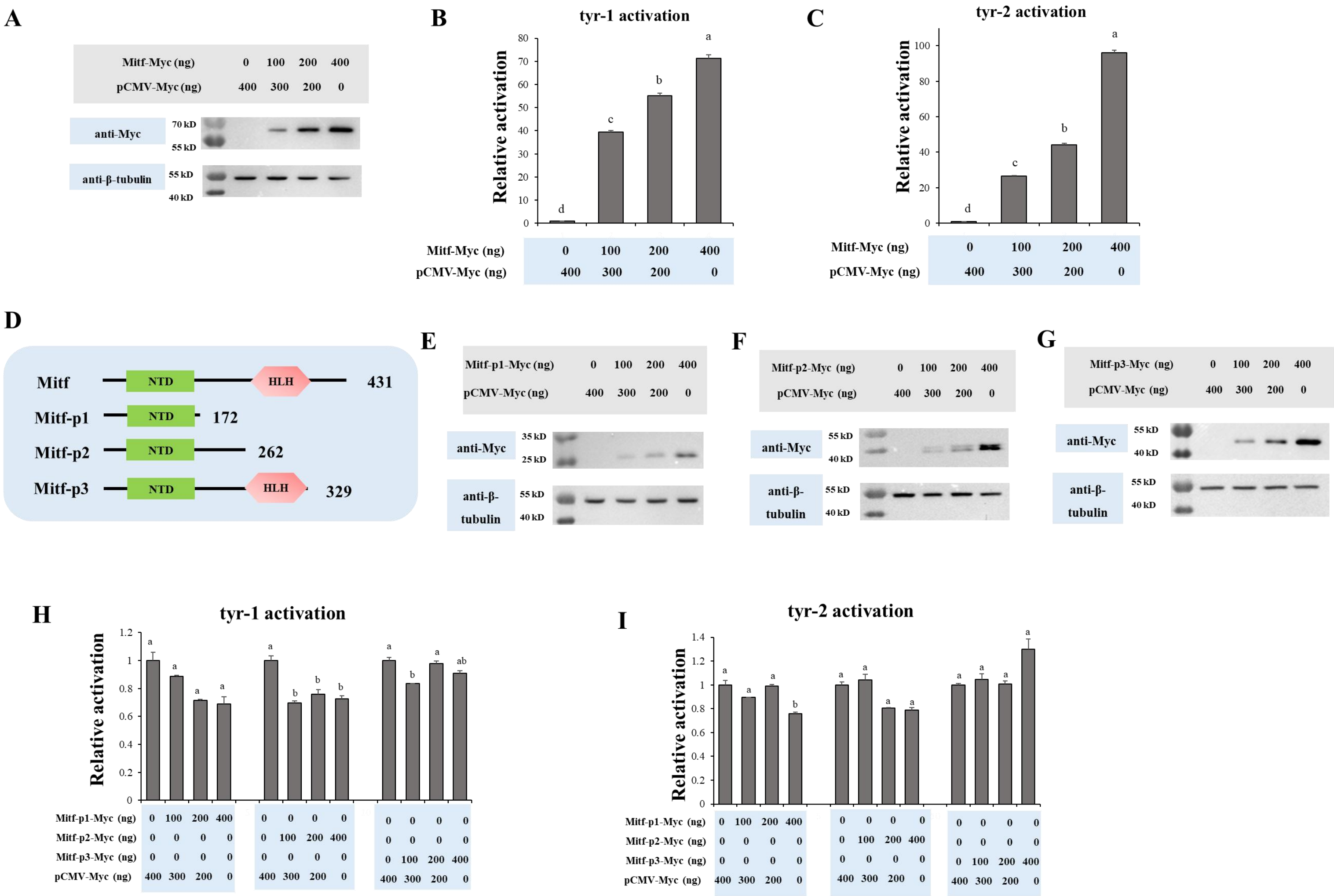

fig. S14. Regulation of *tyr* promoters by full-length MITF and truncated MITF mutants.

(A) Western blot showing dose-dependent expression of full-length MITF-Myc in HEK293T cells used for the dual-luciferase reporter assays. Cells were transfected with increasing amounts of *mitf*-Myc plasmid (0, 100, 200, and 400 ng), while the amount of empty pCMV-Myc vector was adjusted accordingly to keep the total plasmid amount constant. *β*-tubulin was used as the loading control. (B and C) Dual-luciferase reporter assays showing dose-dependent activation of the *tyr-1* (B) and *tyr-2* (C) promoters by full-length MITF. Firefly luciferase activity was normalized to *Renilla* luciferase activity. (D) Schematic structures of full-length MITF and three truncated mutants, MITF-p1, MITF-p2, and MITF-p3. NTD, N-terminal domain; HLH, helix-loop-helix domain. (E-G) Western blots showing dose-dependent expression of MITF-p1-Myc (E), MITF-p2-Myc (F), and MITF-p3-Myc (G), with *β*-tubulin used as the loading control. (H and I) Dual-luciferase reporter assays testing the effects of MITF truncated mutants on *tyr-1* (H) and *tyr-2* (I) promoter activity. Data are shown as mean ± SEM; $n = 3$ biological replicates. Statistical significance was assessed using one-way ANOVA followed by Tukey's HSD post hoc test. Different letters indicate significant differences at $P < 0.05$.

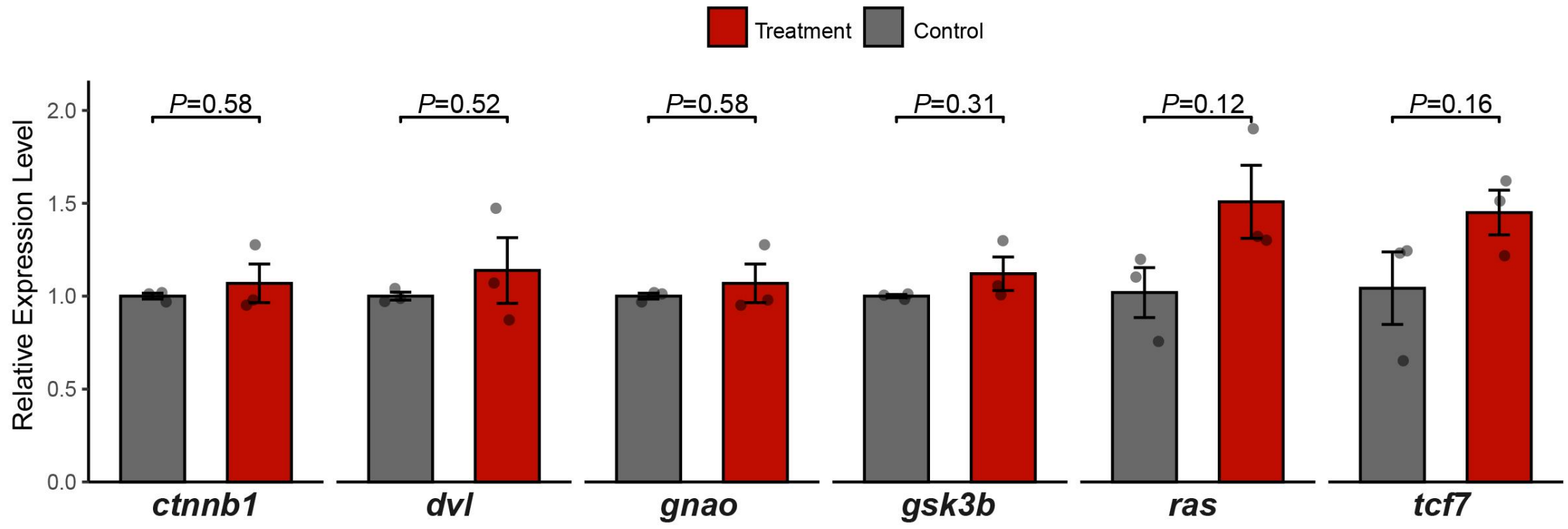

fig. S15. Expression of upstream or pathway-related genes after *mitf* RNAi in *Haliotis iris*. qRT-PCR analysis of *ctnnb1, dvl, gnao, gsk3b, ras,* and *tcf7* expression in mantle tissue after *mitf* knockdown. Relative expression was calculated using the $2^{-\Delta\Delta Ct}$ method and normalized to the control group. Data are shown as mean ± SEM; $n = 3$ biological replicates per group. Statistical significance was assessed using independent-samples *t* tests, with *P* values indicated above the corresponding comparisons. None of these upstream or pathway-related genes showed significant downregulation after *mitf* RNAi, supporting that the reduced expression of *tyr-1* and *tyr-2* was not due to general non-specific transcriptional suppression.

Tables included as Excel spreadsheets

table S1. Summary statistics of sequencing data used for genome assembly, transcriptomic, chromatin-interaction, methylome, and developmental analyses of *Haliotis iris*. This table lists the sequencing type, read count, data size, sequencing depth, sample name, and tissue or developmental stage for each dataset used in this study. Depth was calculated as total data size divided by the assembled genome size; for RNA-seq libraries, it represents sequencing data yield rather than genomic coverage. Sample names correspond to those used in the figures, supplementary figures, and downstream analyses.

table S2. BUSCO completeness assessment of the *Haliotis iris* genome assembly and predicted protein set. Genome assembly completeness and annotated protein-set completeness were evaluated using BUSCO with the metazoa_odb10 dataset.

table S3. Repetitive element annotation of the rainbow abalone genome. Repetitive elements in the rainbow abalone genome were annotated using a combination of *de novo* and homology-based approaches, including RepeatModeler, RepeatMasker, and RepeatProteinMask. Length indicates the total genomic span assigned to each repeat category, and percent of genome indicates the proportion relative to the final assembled genome size. The total columns represent the non-redundant repeat annotation after merging overlapping repeat intervals across the different annotation approaches.

table S4. Summary of protein-coding gene annotation and mantle-expressed non-coding transcripts in the *Haliotis iris* genome. Total supported genes represents the non-redundant union of genes supported by either public database annotation or transcript evidence. Percentages for protein-coding genes were calculated relative to the total number of predicted protein-coding genes in the genome (20,994).

table S5. Synteny matrix between extant gastropod chromosomes and inferred

ancestral proto-chromosomes. This table shows the manually curated synteny matrix underlying Fig. 1C. Rows represent chromosomes of the six representative gastropod species, columns represent inferred ancestral proto-chromosomes of Orthogastropoda and Apogastropoda, and values indicate the number of syntenic anchors assigned to each chromosome-proto-chromosome pair. These assignments were used to visualize ancestral proto-chromosomal fragments and chromosomal rearrangements in Fig. 1C.

table S6. Ecological and life-history context for karyotype evolution in the 18 gastropod species included in fig. S5. This table summarizes habitat, inferred dispersal context, and relative karyotype status for the 18 gastropod species used in the expanded chromosomal comparison. Karyotype status was assessed relative to the conservation or rearrangement of ancestral proto-chromosomal fragments shown in fig. S5. Key references supporting habitat, life-history, dispersal, and taxonomic information are listed for each species. Where species-specific larval biology was unavailable, dispersal context was inferred cautiously from taxonomic databases, species accounts, and classical malacological literature.

table S7. Differential expression analysis of protein-coding genes between paired left and right cephalic tentacles. The top 10 genes ranked by nominal *P* value are listed. No genes passed the FDR-adjusted significance threshold in this comparison. The adjusted *P* value (*P*adj) was used as the primary criterion for statistical significance. BaseMean, $\log_2$FoldChange, *P* value, and *P*adj are reported from the DESeq2 differential expression analysis based on read counts, whereas sample-level expression values for the left and right cephalic tentacles are shown as TPM. Information corresponding to the sample IDs in each group is provided in table S1.

table S8. Differentially expressed genes between the asymmetric mantle lobes before and after excluding the relatively separated individual in PCA. Group R1 represents the analysis using all four individuals, whereas group R2 represents the reanalysis after excluding individual 1, whose paired mantle samples (1-ZW-1 and

1-YW-1) showed relatively separated clustering in the PCA analysis (fig. S6A). For each analysis, the top 10 genes ranked by nominal *P* value are listed. The FDR-adjusted *P* value (*P*adj) was used as the primary criterion for statistical significance. Sample-level columns show normalized expression (TPM) values for the left mantle lobe (ZW) and right mantle lobe (YW). Information corresponding to the sample IDs in each group is provided in table S1.

table S9. Raw qRT-PCR data for *pitx* expression in Fig. 2B and *lncRNA1/lncRNA2* expression in Fig. 2D in *Haliotis iris*. L and R indicate paired left and right mantle-lobe samples from the same individual, respectively. A total of 16 paired biological replicates were analyzed. For each target gene, duplicate Ct values and their averages are shown. *GAPDH* was used as the reference gene for normalization. Relative expression levels were calculated using the $2^{-\Delta\Delta Ct}$ method.

table S10. Raw qRT-PCR data for *pitx* expression in paired left and right mantle lobes of four additional molluscan species. This table provides the raw qRT-PCR data of *pitx* expression analysis shown in Fig. 2B, including *Haliotis discus*, *Scapharca broughtonii*, *Crassostrea gigas*, and *Mizuhopecten yessoensis*. L and R indicate paired left and right mantle-lobe samples, respectively. Sample sizes were n = 8 biological replicates for *H. discus*, n = 9 for *S. broughtonii*, n = 14 for *C. gigas*, and n = 8 for *M. yessoensis*. Species-specific reference genes were used for normalization as indicated in the column headers: *β-actin* for *H. discus* and *M. yessoensis*, *EF-1α* for *S. broughtonii*, and *RS18* for *C. gigas*. Relative expression levels were calculated using the $2^{-\Delta\Delta Ct}$ method. Statistical significance in Fig. 2B was assessed using paired *t* tests. *n* indicates the number of biological replicates.

table S11. Differential expression of ncRNAs between paired left and right mantle lobes and cephalic tentacles before and after excluding the relatively separated mantle individual in PCA. For the mantle-lobe comparison, Asymmetric mantle lobes-R1 represents the analysis using all four individuals, whereas Asymmetric mantle lobes-R2 represents the reanalysis after excluding individual 1,

whose paired mantle samples (1-ZW-1 and 1-YW-1) showed relatively separated clustering in the PCA analysis (fig. S6A). No ncRNAs showed statistically significant differential expression between the symmetric cephalic tentacles. In the R1 mantle-lobe analysis, only *lncRNA1* and *lncRNA2* showed significant differential expression; in the R2 mantle-lobe analysis, only *lncRNA1* remained statistically significant. For *lncRNA2* in the R2 analysis, sample 2-ZW-1 was diagnosed as an outlier and removed, leaving only two biological replicates; therefore, no reliable P value or FDR-adjusted P value (Padj) was obtained. The Padj value was used as the primary criterion for statistical significance. Information corresponding to the sample IDs in each group is provided in table S1.

table S12. Raw qRT-PCR data for reciprocal RNAi assays of *lncRNA1*, *lncRNA2*, and *pitx* in *Haliotis iris*. This table provides the raw qRT-PCR data used for the RNAi analyses shown in Fig. 2E and fig. S8. NC indicates the negative-control RNAi group, and RNAi indicates samples treated with siRNAs targeting the indicated gene. For each sample, duplicate Ct values and their averages are shown for *pitx*, *lncRNA1*, *lncRNA2*, and the reference gene *GAPDH*. Relative expression levels were calculated using the $2^{-\Delta\Delta Ct}$ method. For *lncRNA1* and *lncRNA2* RNAi experiments, $n = 9$ biological replicates per group; for the *pitx* RNAi experiment, $n = 3$ biological replicates per group. Statistical significance in the corresponding figures was assessed using independent-samples *t* tests.

table S13. Probe sequences used in RNA localization and pulldown assays. All sequences are shown in the 5’ to 3’ orientation. For reciprocal miRNA pulldown-qRT-PCR probes, “5’-biotin” indicates a 5’ biotin modification used for streptavidin-based capture. Random probe 1 and random probe 2 were mixed at a 1:1 ratio and used as scrambled controls, and miRNA1 and miRNA2 probes were mixed at a 1:1 ratio for the miRNA1/2 pulldown group. Species-specific *pitx* and *lncRNA1* probes were designed for *Monodonta labio* and *Cellana toreuma in situ* hybridization. The Location column indicates the figure panels in which each probe was used.

table S14. The information of miRNAs captured by the HyPro-seq assay using the *lncRNA1* probe. Candidate miRNAs with a read count ≥5 in the HyPro-seq library were retained. The input library represents the total miRNA profile generated by direct small RNA sequencing of the same experimental tissue without HyPro-proximity labeling or probe-based pull-down, and was used to estimate the endogenous miRNA background. Candidate miRNAs captured by the *lncRNA1* HyPro pull-down were annotated by homology searches against the reference miRNA sequences of the *Haliotis rufescens* in MirGeneDB 3.0 (https://www.mirgenedb.org/). Only the top 30 miRNAs ranked by read count in the HyPro library were assigned Study_IDs. A dash indicates that no confident homologous hit or annotation was identified in MirGeneDB 3.0. All coordinates comply with the GFF-style coordinate convention.

table S15. Raw qRT-PCR data for *pitx* and *lncRNA1* enrichment in the reciprocal miRNA pulldown-qRT-PCR assay shown in Fig. 3E. SiRS18, the *RS18* gene from *Strongylocentrotus intermedius*, was used as the normalization reference gene. The scrambled control consisted of two random probes mixed at a 1:1 ratio, and the miRNA1/2 group consisted of miRNA1 and miRNA2 probes mixed at a 1:1 ratio. Information for miRNA1, miRNA2, miRNA3, and miRNA5 is provided in table S14.

table S16. TargetScan prediction of canonical miRNA seed matches between candidate miRNAs and *pitx* or *lncRNA1*. The Seed column indicates the miRNA seed sequence used for target-site prediction. Four canonical TargetScan site classes were examined: 6mer, 7mer-A1, 7mer-m8, and 8mer sites. Match_seq indicates the sequence in the target transcript that pairs with the miRNA seed region. Start/End indicate the position of the Match_seq in the mature transcript, numbered from the first nucleotide of the transcript. Hyphens indicate that no corresponding canonical seed-matching site was predicted.

table S17. Genomic coordinates of synteny-supported candidate *lncRNA1* loci in three representative gastropod species. The ncRNA ID corresponds to the transcript

identifier used in the comparative synteny analysis shown in Fig. 4C. Chromosome indicates the genomic scaffold or chromosome on which the candidate ncRNA is located. Location_start and Location_end indicate the genomic coordinate interval of the transcript, and Strand indicates its transcriptional orientation. Because lncRNAs can evolve rapidly at the sequence level, these loci were identified primarily based on their transcript evidence and conserved local genomic association with *pitx* rather than deep sequence conservation alone.

table S18. Raw qRT-PCR data for *pitx* and *lncRNA1* expression in paired left and right mantle lobes of *Cellana toreuma* and *Monodonta labio*. This table provides the raw qRT-PCR data used for the cross-species left-right expression analysis shown in fig. S9A and fig. S9D. L and R indicate paired left and right mantle-lobe samples from the same individual, respectively. Sample sizes were $n = 7$ biological replicates for *M. labio* and $n = 8$ biological replicates for *C. toreuma*. For each sample, duplicate Ct values and their averages are shown for *pitx*, *lncRNA1*, and the reference gene *β-actin*. Relative expression levels were calculated using the $2^{-\Delta\Delta Ct}$ method. Statistical significance in fig. S9A and fig. S9D was assessed using paired *t* tests. *n* indicates the number of biological replicates.

table S19. Raw qRT-PCR data for RNAi assays of *lncRNA1* and *pitx* in *Cellana toreuma* and *Monodonta labio*. This table provides the raw qRT-PCR data used for the cross-species RNAi analyses shown in fig. S9B and fig. S9E. NC indicates the negative-control RNAi group, and RNAi indicates samples treated with siRNAs targeting either *lncRNA1* or *pitx*. For each sample, duplicate Ct values and their averages are shown for *lncRNA1*, *pitx*, and the reference gene *β-actin*. Relative expression levels were calculated using the $2^{-\Delta\Delta Ct}$ method. For *M. labio*, $n = 7$ biological replicates per group; for *C. toreuma*, $n = 6$ biological replicates per group. Statistical significance in fig. S9B and fig. S9E was assessed using independent-samples *t* tests. *n* indicates the number of biological replicates.

table S20. Genes upregulated in pigmented regions in two pigmentation-related

transcriptomic comparisons. This table lists genes upregulated in pigmented regions in either or both of the two transcriptomic comparisons shown in Fig. 5A: pigmented epithelium versus unpigmented muscle (PE1 *vs.* UM) and pigmented epithelium versus unpigmented epithelium (PE2 *vs.* UE). The Group column indicates whether a gene was upregulated in both comparisons ("both"), only in the PE1 *vs.* UM comparison ("PE1"), or only in the PE2 vs. UE comparison ("PE2"). BaseMean, log_2FoldChange, *P* value, and adjusted *P* value (*P*adj) are reported from the DESeq2 differential expression analysis based on read counts. *P*adj was used as the primary criterion for statistical significance. Sample-level expression values are shown as TPM. Information corresponding to the sample IDs is provided in table S1.

table S21. Genes downregulated in pigmented regions in two pigmentation-related transcriptomic comparisons. This table lists genes downregulated in pigmented regions in either or both of the two transcriptomic comparisons shown in Fig. 5A: pigmented epithelium versus unpigmented muscle (PE1 *vs.* UM) and pigmented epithelium versus unpigmented epithelium (PE2 *vs.* UE). The Group column indicates whether a gene was downregulated in both comparisons ("both"), only in the PE1 *vs.* UM comparison ("PE1"), or only in the PE2 *vs.* UE comparison ("PE2"). BaseMean, log_2FoldChange, *P* value, and adjusted *P* value (*P*adj) are reported from the DESeq2 differential expression analysis based on read counts. *P*adj was used as the primary criterion for statistical significance. Sample-level expression values are shown as TPM. Information corresponding to the sample IDs is provided in table S1.

table S22. TPM expression levels of *tyr* paralogs in tissues analyzed in Fig. 5A and Fig. 5I. This table summarizes the transcript abundance of the two *Haliotis iris* *tyr* paralogs, *tyr-1* and *tyr-2*, in the tissues used for the pigmentation-related comparisons in Fig. 5A and the tissue-expression analysis in Fig. 5I. Expression values are shown as TPM. PE1 indicates pigmented lower pedal epithelium from normally pigmented individuals, UM indicates unpigmented lower pedal muscle, PE2

indicates pigmented epithelium from color-variant individuals, and UE indicates unpigmented epithelium from color-variant individuals. “Other type” refers to additional tissues included in the broader tissue-expression survey shown in Fig. 5I. Sample IDs correspond to those listed in table S1.

table S23. Raw dual-luciferase reporter data for evaluating MITF activation of *tyr-1* and *tyr-2* promoters. This table provides the raw luminescence values used for the dual-luciferase reporter assays shown in Fig. 5F. The indicated *mitf* values represent the amount of transfected MITF expression plasmid. Firefly luciferase activity reports *tyr-1* or *tyr-2* promoter activity, and *Renilla* luciferase activity was used as the internal normalization control. Relative luciferase activity was calculated by normalizing firefly luciferase values to the corresponding *Renilla* values and then scaling to the 0-ng *mitf* control group. Each condition included $n = 3$ biological replicates. Statistical significance in Fig. 5F was assessed using independent-samples t tests.

table S24. Raw qRT-PCR data for *mitf* knockdown and *tyr* expression after *mitf* RNAi in *Haliotis iris*. This table provides the raw qRT-PCR data used for the *mitf* RNAi analysis shown in Fig. 5H. NC indicates the negative-control RNAi group, and RNAi indicates samples treated with siRNAs targeting *mitf*. Ct values are shown for the reference gene and each target gene, including *mitf*, *tyr-1*, and *tyr-2*. Relative expression levels were calculated using the $2^{-\Delta\Delta Ct}$ method. Average indicates the mean relative expression level for each group. Data in Fig. 5H are shown as mean ± SEM; $n = 3$ biological replicates per group. Statistical significance was assessed using independent-samples t tests.

table S25. Source data of fig. S15, the raw data (Ct value) of qRT-PCR showing *mitf*-RNAi and the relative expression of other genes. This table provides the raw qRT-PCR data used for the analysis of upstream or pathway-related gene expression after *mitf* RNAi, as shown in fig. S15. NC/control indicates the negative-control RNAi group, and RNAi indicates samples treated with siRNAs targeting *mitf*. Ct

values are shown for the reference gene *β-actin* and the target genes *gnao*, *dvl*, *gsk3b*, *ctnnb1*, *tcf7*, and *ras*. Relative expression levels were calculated using the $2^{-\Delta\Delta Ct}$ method. Data in the corresponding figure are shown as mean ± SEM; n = 3 biological replicates per group. Statistical significance was assessed using independent-samples t tests.

table S26. DeepTMHMM-based transmembrane topology prediction of selected SLC26 family proteins. This table summarizes predicted transmembrane regions (TMRs) and membrane topology for selected SLC26 family proteins, including *prestin/slc26a5* homologs and related *slc26a5-like* proteins. Transmembrane topology was predicted using DeepTMHMM after sequence alignment to a 908-amino-acid coordinate framework. The Species and Sequence ID columns indicate the source species and corresponding protein or gene identifier. The Type column indicates the predicted topological state: TMhelix, transmembrane helix; inside, cytoplasmic-facing region; outside, extracellular or luminal-facing region. Start and End indicate the amino-acid coordinate interval of each predicted topological segment in the aligned sequence. The number of predicted TMRs is indicated for each protein block.

table S27. Expression responses of *prestin* and additional control genes in mantle tissue after vibration treatment in Fig. 6I. qRT-PCR analysis of *prestin1*, *prestin2*, and selected non-target genes after simulated vibration treatment. Both *prestin1* and *prestin2* were significantly upregulated in the treatment group relative to the control group, whereas no significant changes were detected for the other examined genes (*coadhesin1*, *coadhesin2*, *spondin*, *dvl*, *tcf7*, *gsk3b*, *wnt*, *mitf*, *jun*, *hsp90*, and *hsp40*). These results indicate a selective transcriptional response of the mantle *prestin-like* genes to vibration stimulation. Relative expression levels were calculated using the $2^{-\Delta\Delta Ct}$ method. Data in the corresponding figure are shown as mean ± SEM; n = 8 biological replicates per group. Statistical significance was assessed using independent-samples t tests.

table S28. Twelve additional gastropod genomes used in the expanded

chromosome-evolution analysis. This table summarizes the 12 additional chromosome-level or near chromosome-level gastropod genomes included in the expanded chromosomal comparison shown in fig. S5. Together with the six representative genomes used in Fig. 1C, these genomes were used to assess the distribution of ancestral proto-chromosomal fragments across 18 gastropod species. Genome size, NCBI assembly accession, number of chromosomes, and BUSCO completeness scores for genome assemblies and predicted protein sets are listed. BUSCO assessments were performed using the mollusca_odb10 dataset.

table S29. Primer and oligonucleotide sequences used in this study. All sequences are shown in the 5' to 3' orientation. For qRT-PCR primers, "qF" and "qR" indicate forward and reverse primers, respectively. For RNAi oligonucleotides, sense and antisense strands are listed separately. The application column indicates the experimental use of each primer or oligonucleotide, and the location column indicates the corresponding figure or supplementary figure in which the primer set was used.

table S30. Public gastropod transcriptome datasets used to support candidate *lncRNA1* annotation in the comparative synteny analysis. This table lists the publicly available gastropod transcriptome datasets downloaded from NCBI and used to support transcript annotation and comparative synteny analyses. Species, BioProject accessions, SRA experiment accessions, and sample or tissue names are provided for each dataset. These transcriptome data were used to help identify expressed transcripts in the corresponding genomic regions, including candidate ncRNA loci associated with the *pitx*-linked syntenic region. The Name column indicates the sample or tissue label provided in the original NCBI record.
